# Monovalent lanthanide(I) in borozene complexes

**DOI:** 10.1038/s41467-021-26785-9

**Published:** 2021-11-09

**Authors:** Wan-Lu Li, Teng-Teng Chen, Wei-Jia Chen, Jun Li, Lai-Sheng Wang

**Affiliations:** 1 Department of Chemistry and Key Laboratory of Organic Optoelectronics & Molecular Engineering of Ministry of Education, Tsinghua University, 100084 Beijing, China; 2 Department of Chemistry, Brown University, Providence, RI 02912 USA; 3 Department of Chemistry, Southern University of Science and Technology, 518055 Shenzhen, China

**Keywords:** Chemical bonding, Physical chemistry, Chemical physics, Computational chemistry

## Abstract

Lanthanide (Ln) elements are generally found in the oxidation state +II or +III, and a few examples of +IV and +V compounds have also been reported. In contrast, monovalent Ln(+I) complexes remain scarce. Here we combine photoelectron spectroscopy and theoretical calculations to study Ln-doped octa-boron clusters (LnB_8_^−^, Ln = La, Pr, Tb, Tm, Yb) with the rare +I oxidation state. The global minimum of the LnB_8_^−^ species changes from *C*_s_ to *C*_*7v*_ symmetry accompanied by an oxidation-state change from +III to +I from the early to late lanthanides. All the *C*_*7v*_-LnB_8_^−^ clusters can be viewed as a monovalent Ln(I) coordinated by a η^8^-B_8_^2−^ doubly aromatic ligand. The B_7_^3−^, B_8_^2−^, and B_9_^−^ series of aromatic boron clusters are analogous to the classical aromatic hydrocarbon molecules, C_5_H_5_^−^, C_6_H_6_, and C_7_H_7_^+^, respectively, with similar trends of size and charge state and they are named collectively as “borozenes”. Lanthanides with variable oxidation states and magnetic properties may be formed with different borozenes.

## Abstract (web summary)

The most common oxidation state for lanthanides is +3. Here the authors use photoelectron spectroscopy and theoretical calculations to study half-sandwich complexes where a lanthanide center in the oxidation state +1 is bound to an aromatic wheel-like B_8_^2-^ ligand.

## Introduction

Oxidation state (OS) is a fundamental chemical concept^1^. The discovery of new and unusual OS for chemical elements has drawn persistent attention in chemistry and materials science. The OS of lanthanide elements has been of particular interest because it is directly related to the unique chemical, magnetic, and optical properties of lanthanide compounds^2,3^. Lanthanides were considered usually to exist mainly in the stable +III OS due to the chemical inertness of the 4 *f* electrons. Recent studies have provided evidence that all lanthanides can form divalent complexes^4–11^, whereas stable tetravalent compounds are known only for a few lanthanides^12–16^. The highest oxidation state known for lanthanides is +V, observed recently in gaseous species, PrO_4_, PrO_2_^+^, and NPrO^17,18^. However, monovalent Ln(I) species are quite rare. The lanthanide iodide (LaI) synthesized by heating LaI_3_ with metallic lanthanum has the nominal La(I) OS, but was shown to contain La–La metallic bonding^19^. Besides gas-phase diatomic lanthanide hydride and halide molecules^20–22^, the only other previous example of Ln(I) is the PrB_4_^−^ [i.e., (Pr^I^)(B_4_^2−^)] cluster characterized by photoelectron spectroscopy (PES) and quantum chemistry calculations^23^. The rare earth Sc element, which is in the same group as La, was known to have Sc(I) OS in multi-decker molecular compounds^24,25^. Compounds with low OS lanthanides will not only expand the chemistry of the lanthanide elements, but can also potentially serve as strong reducing agents in organometallic syntheses^26–28^. It would be interesting to discover suitable ligands that can stabilize monovalent lanthanides.

Joint PES and quantum chemistry studies over the past decade have shown that size-selected anionic boron clusters (B_*n*_^−^) are planar over a wide size range, stabilized by *σ* and *π* double aromaticity^29–33^. The *π* bonding patterns of many planar boron clusters are analogous to polycyclic aromatic compounds^34,35^. One of the first boron clusters found to exhibit *σ* and *π* double aromaticity was the wheel-like *D*_*8h*_ B_9_^−^, which satisfies the (4 *N* + 2) Hückel rule with *N* = 1 for both the delocalized *σ* and *π* electrons^36^. The *D*_*8h*_ B_9_^−^ cluster inspired the design and characterization of a new class of borometallic molecular wheels (M©B_*n*_^−^, *n* = 8–10) with double aromaticity^37–40^. Several small mono-lanthanide boron clusters have been studied^23,41–43^. Specifically, the PrB_7_^−^ cluster was shown to form a half-sandwich structure, in which a Pr(II) center was coordinated by an aromatic η^7^-B_7_^3−^ ligand^41^. Recently, a series of di-lanthanide boron clusters Ln_2_B_*n*_^−^ (*n* = 7–9) were found to form inverse sandwich complexes with boron monocyclic rings^44,45^. An interesting question is if mono-lanthanide boron clusters would form Ln©B_*n*_^−^ type of molecular wheels, similar to the mono-transition-metal boron clusters^37–40^.

Here we report a PES and quantum chemistry study on a series of lanthanide-doped octa-boron clusters, LnB_8_^−^ (Ln = La, Pr, Tb, Tm, Yb). Instead of the Ln©B_8_^−^ wheel-like structures, we find two types of structures for Ln = La and Pr: a *C*_*s*_ three-dimensional (3D) global minimum and a low-lying co-existing *C*_*7v*_ half-sandwich structure, whereas the latter becomes the global minimum for the late lanthanides (Tb, Tm, and Yb). The *C*_*s*_ 3D LnB_8_^−^ clusters contain a Ln(III) center, but the *C*_*7v*_ structures all contain a Ln(I) center coordinated by a η^8^-B_8_^2−^ doubly aromatic ligand. We found that the frontier orbitals of the B_8_^2−^ ligand match favorably with the Ln 5*d* orbitals to afford strong metal-ligand chemical bonding. The B_8_^2−^ ligand is shown to be one member of a series of doubly aromatic planar boron clusters, B_7_^3−^, B_8_^2−^, and B_9_^−^, which are analogous to the aromatic C_5_H_5_^−^, C_6_H_6_, and C_7_H_7_^+^ hydrocarbons, respectively. This series of aromatic boron ligands provides the possibility to design lanthanide boride complexes with tunable OS and magnetic properties.

## Results and discussion

### Photoelectron spectroscopy

**Fig. 1:**
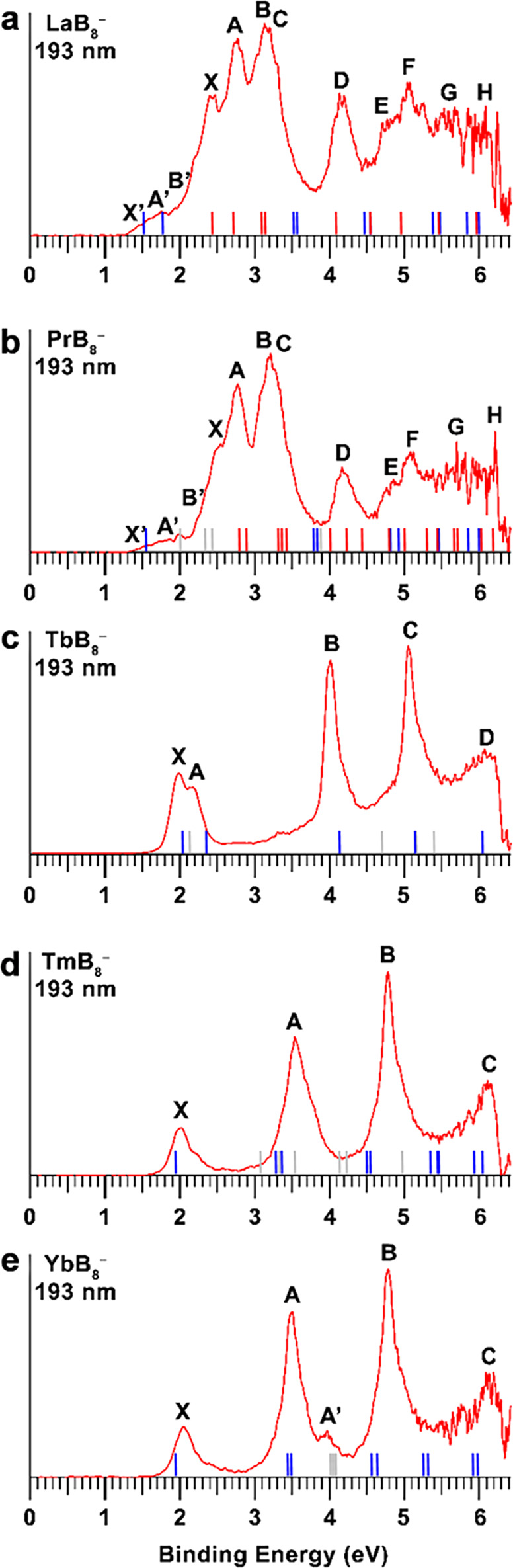
Photoelectron spectra at 193 nm (6.424 eV) of LnB_8_^−^ (Ln = La, Pr, Tb, Tm, Yb). **a** LaB_8_^−^. **b** PrB_8_^−^. **c** TbB_8_^−^. **d** TmB_8_^−^. **e** YbB_8_^−^. The blue and red bars in **a** and **b** represent computed VDEs of isomer I (*C*_*7v*_) and isomer II (*C*_*s*_), respectively. The gray bars indicate the calculated 4*f*-detachment channels.

We conducted the PES experiments using a home-built magnetic-bottle apparatus, which consisted of a laser vaporization cluster source and a time-of-flight mass spectrometer (Methods and Supplementary Fig. 1 for more details)^31^. The LnB_8_^−^ (Ln = La, Pr, Tb, Tm, Yb) clusters were generated by laser ablation of a disk target consisting of Ln and isotopically enriched ^11^B. The clusters were entrained by a helium carrier gas (containing 5% argon) and underwent a supersonic expansion. Negative ions were extracted from the cluster beam perpendicularly and separated by the time-of-flight mass spectrometer. The octa-boron clusters (LnB_8_^−^) were selected and decelerated before photodetachment. Two photon energies were used in the current study, including the third harmonic of a Nd:YAG laser (355 nm, 3.496 eV) and the 193 nm (6.424 eV) radiation from an ArF excimer laser. Photoelectrons were analyzed by the magnetic-bottle electron analyzer and calibrated using the Bi^−^ atomic spectrum. Photoelectron spectra of LnB_8_^−^ (Ln = La, Pr, Tb, Tm, Yb) at 193 nm are presented in Fig. 1, and the 355 nm spectra of LaB_8_^−^, PrB_8_^−^, and YbB_8_^−^ are given in Supplementary Fig. 2. The PES bands are designated with letters (X, A, B, …), and the vertical detachment energies (VDEs) measured from the maxima of the observed bands are given in Supplementary Tables 1–5 for LaB_8_^−^, PrB_8_^−^, TbB_8_^−^, TmB_8_^−^, and YbB_8_^−^, respectively. Based on the observed spectral patterns, the five species can be divided into three groups, (1) LaB_8_^−^ and PrB_8_^−^, (2) TbB_8_^−^, and (3) TmB_8_^−^ and YbB_8_^−^.

The photoelectron spectra of the two early-lanthanide octa-boron clusters (LaB_8_^−^ and PrB_8_^−^) are similar, suggesting they should have similar structures and bonding. Both spectra display complicated spectral patterns with congested PES bands, most likely due to the existence of multiple isomers. In the low binding energy region of LaB_8_^−^, we observed four intense bands (X, A, B/C), where bands B and C overlapped and were only resolved in the 355 nm spectrum (Supplementary Fig. 2a). Band X should be the ground state transition of the major isomer, yielding a VDE_1_ of 2.40 eV and an estimated adiabatic detachment energy (ADE) of ~2.2 eV, which also represents the electron affinity (EA) of neutral LaB_8_. The VDE of the intense band A was measured to be 2.77 eV, whereas those of B and C were found to be 2.99 and 3.18 eV, respectively, from the 355 nm spectrum (Supplementary Fig. 2a). Following an energy gap, a well-resolved band D at 4.14 eV was observed. Beyond band D, almost continuous spectral features were observed. Bands E, F, G, and H were tentatively labeled for the sake of discussion. The broad weak features (X’, A’, B’) on the low binding energy side suggested the co-existence of low-lying isomers for LaB_8_^−^ in the cluster beam. This part of the spectrum was resolved slightly better in the 355 nm spectrum (Supplementary Fig. 2a). Bands X’ and A’ were broad with VDEs of ~1.5 eV and 1.9 eV, respectively, while band B’ at 2.16 eV was better defined. Higher binding energy transitions of this isomer were likely buried in the signals of the main isomer. The observed features and binding energies for LaB_8_^−^ are given in Supplementary Table 1, where they are compared with the calculated values. The photoelectron spectra of PrB_8_^−^ are almost identical to those of LaB_8_^−^ (Fig. 1 and Supplementary Fig. 2); the observed spectral features and their binding energies are given in Supplementary Table 2, along with the calculated values.

The 193 nm spectrum of TbB_8_^−^ has a much simpler pattern in comparison to those of LaB_8_^−^ and PrB_8_^−^, with four clearly resolved bands. The lowest-binding energy peak X gives rise to the first VDE at 1.98 eV and an ADE of 1.87 eV, followed by a close-lying band A at 2.18 eV. After a large energy gap of ~2 eV, two sharp and intense bands are displayed: band B at 4.02 eV and C at 5.06 eV. Beyond 5.5 eV, the spectrum becomes nearly continuous and a band D at around 6.1 eV is tentatively assigned. The binding energies of all the observed bands are given in Supplementary Table 3.

The spectrum of TmB_8_^−^ displays the simplest spectral pattern with four clearly resolved bands: X, A, B, and C. Band X with a VDE of 2.02 eV is well resolved and an ADE of 1.90 eV is evaluated from its onset. Following a large energy gap of about 1.5 eV, band A at 3.54 eV is broader and more intense, which may contain multiple detachment channels. Following another large energy gap of 1.2 eV, a sharp and intense band B is observed at 4.79 eV. The fourth band C is observed at the high binding energy side with a VDE of 6.1 eV. The spectrum of YbB_8_^−^ is nearly identical to that of TmB_8_^−^ except that a weak feature (A’) were resolved around the second main PES band. The binding energies of the observed PES bands for TmB_8_^−^ and YbB_8_^−^ are given in Supplementary Tables 4 and 5, respectively. The simple spectral patterns of TmB_8_^−^ and YbB_8_^−^ suggest their structures must be highly symmetric. The spectrum of TbB_8_^−^ is more like those of the late lanthanides (TmB_8_^−^ and YbB_8_^−^) than the early lanthanides (LaB_8_^−^ and PrB_8_^−^), indicating TbB_8_^−^ may have a similar structure as those of TmB_8_^−^ and YbB_8_^−^.

### Global minimum structural searches

**Fig. 2:**
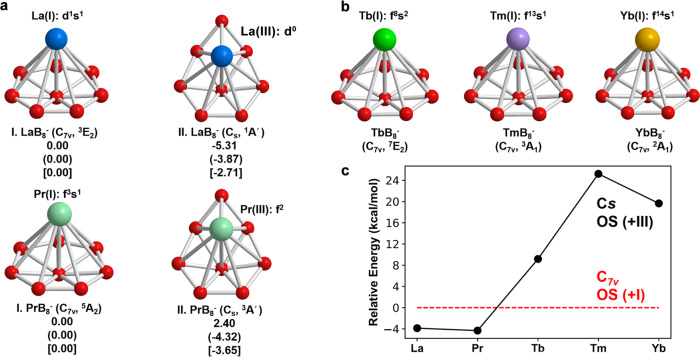
The structures of LnB_8_^−^ (Ln = La, Pr, Tb, Tm, Yb). **a** The global minima and low-lying isomers of LaB_8_^−^ and PrB_8_^−^ at the PBE, PBE0 (in parenthesis), CCSD(T) (in bracket) levels, with each corresponding electronic configuration. **b** The global minima of TbB_8_^−^, TmB_8_^−^, and YbB_8_^−^. **c** The energy difference between the LnB_8_^−^ structures with the +III OS (3D *C*_*s*_ structure) and +I OS (*C*_*7v*_ structure) at the PBE0/TZP level, with the +I OS isomer as the reference.

The global minima for LnB_8_^−^ (Ln = La, Pr, Tb, Tm, Yb) and their low-lying isomers in the cases of LaB_8_^−^ and PrB_8_^−^ are shown in Fig. 2a and b. More isomers within 50 kcal mol^−1^ for LaB_8_^−^ and 65 kcal mol^−1^ for YbB_8_^−^ are shown in Supplementary Figs. S3 and S4, respectively. At the PBE/TZP level, the most stable structure for LaB_8_^−^ is found to be the 3D isomer II (*C*_*s*_, ^1^*A*’), with the half-sandwich isomer I (*C*_*7v*_, ^3^*E*_2_) being 5.31 kcal mol^−1^ higher in energy. At the PBE0/TZP and CCSD(T)/Def2-TZVP levels, the 3D isomer II is still the global minimum. At the more accurate CCSD(T) level, the half-sandwich isomer I is only 2.71 kcal mol^−1^ higher in energy than the 3D isomer II, suggesting that it may be present in the experiment as a minor component. Two similar low-lying isomers are found for PrB_8_^−^; and they are within 4 kcal mol^−1^ in energy at the PBE/TZP, PBE0/TZP, and CCSD(T)/Def2-TZVP levels. Thus, for LaB_8_^−^ and PrB_8_^−^ both the 3D isomer I and the half-sandwich isomer II are close in energy and could co-exist under our experimental conditions. For the late lanthanide LnB_8_^−^ (Ln = Tb, Tm, Yb), the half-sandwich *C*_*7v*_ structure is found to be the global minimum at all levels of theory, with high stabilities over other isomers (Fig. 2c and Supplementary Fig. 4). As will be shown below, the OS of the Ln atoms in the half-sandwich *C*_*7v*_ structure is +I, whereas that in the *C*_*s*_ 3D structures of LaB_8_^−^ and PrB_8_^−^ is +III. The second isomer of the three late lanthanide octa-boron clusters is similar to the *C*_*s*_ 3D isomer II of LaB_8_^−^ and PrB_8_^−^, but they are much higher in energy (Fig. 2c and Supplementary Fig. 4). The relative stabilities of the +I OS structures and the +III OS isomers are exhibited in Fig. 2c for the five lanthanide octa-boron clusters. The coordinates of the global minima of LnB_8_^−^ and the *C*_*7v*_ low-lying isomers for LaB_8_^−^ and PrB_8_^−^, as well as their corresponding neutrals are given in Supplementary Table 6.

### Comparison between experiment and theory

The VDEs of the global minima and low-lying isomers for the LnB_8_^−^ clusters were calculated (see Methods) and compared with the experimental results in Fig. 1 and Supplementary Tables 1–5, respectively. Different levels of theory were used to calculate the VDE_1_ and ADE values for the *C*_*7v*_ structures of all LnB_8_^−^ and the *C*_*s*_ structures for LaB_8_^−^ and PrB_8_^−^ (see Supplementary Table 7). We found that the different levels of theory yielded similar VDE_1_ and ADE values, which all agree well with the measured values.

The global minima for both LaB_8_^−^ and PrB_8_^−^ are found to be the 3D isomer II with *C*_*s*_ symmetry at the CCSD(T)/Def2-TZVP level, while the half-sandwich *C*_*7v*_ structure is a low-lying isomer (Fig. 2a and c). The structures and photoelectron spectra of PrB_8_^−^ and LaB_8_^−^ are nearly identical, because of the nonbonding nature of the highly contracted 4 *f* orbitals and the low detachment cross-sections of *f*-electrons^41,44,46–49^. Thus, we will only discuss LaB_8_^−^ in detail as a representative of the early-lanthanide octa-boron clusters. The computed VDE_1_/ADE for the *C*_*s*_ isomer II of LaB_8_^−^ is 2.47/2.25 eV at the CCSD(T)/Def2-TZVP level (Supplementary Table 7), in excellent agreement with the experimental value of 2.40/2.19 eV. Higher detachment channels of the *C*_*s*_ isomer are complicated as shown in Supplementary Table 1, in good accord with the congested experimental features (Fig. 1a and Supplementary Table 1).

The calculated VDE_1_/ADE for the *C*_*7v*_ isomer I of LaB_8_^−^, 1.47/1.41 eV at the CCSD(T) level (Supplementary Table 7), are much lower than those of isomer II, agreeing well with the weak feature X’ at ~1.5 eV. The first electron detachment is from the singly occupied 3*a*_1_ orbital (primarily of La 6*s* character), as can be seen in Supplementary Table 1 and Supplementary Figs. 5 and 6. The second VDE for the *C*_*7v*_ isomer, corresponding to detachment of the single 1*e*_2_ (La 5*d*_*δ*_) electron (Supplementary Figs. 5 and 6), was calculated to be 1.79 eV (Supplementary Table 1), consistent with the weak peak A’ observed experimentally. The weak band B’ in PrB_8_^−^ is due to detachment from the 4*a*_1_ orbital (Pr 4 *f*_*σ*_) (Supplementary Table 2 and Supplementary Fig. 7). However, the 4*a*_1_ orbital of 4*f* character is not occupied in LaB_8_^−^. The very similar B’ band in LaB_8_^−^ (Supplementary Fig. 2) could be contributed from other competitive electronic states (Supplementary Table 8) due to the strong electron correlation effects. Higher detachment transitions for the *C*_*7v*_ isomer would be buried in the congested spectral features of the main *C*_*s*_ isomer. Overall, the complicated and congested experimental spectra of LaB_8_^−^ and PrB_8_^−^ can be well explained by the global minimum *C*_*s*_ structure as the major species and the *C*_*7v*_ structure as a minor co-existing isomer.

**Fig. 3:**
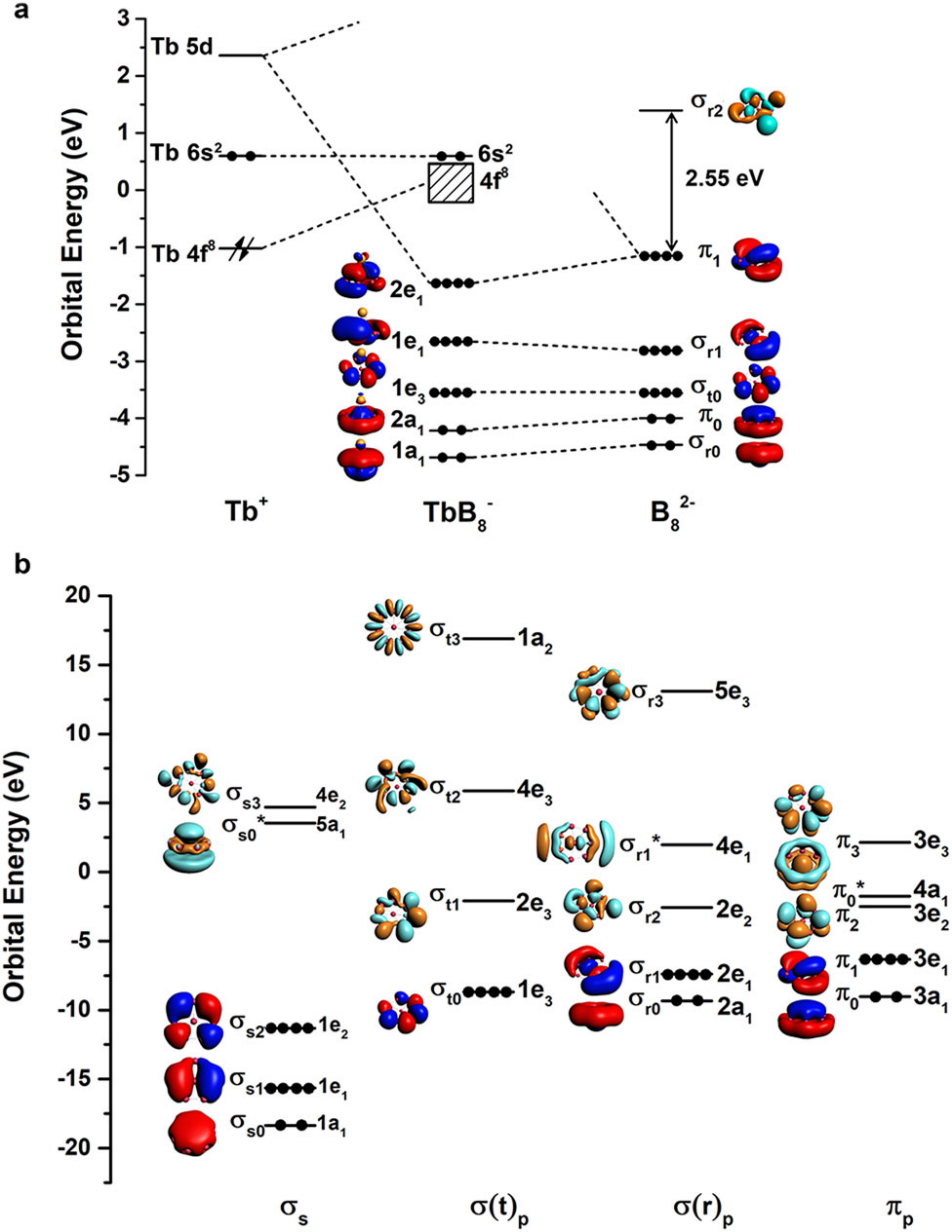
Chemical bonding and orbital interactions. **a** Orbital correlation diagram of the *C*_*7v*_ TbB_8_^−^ with those of Tb^+^ (4 *f*^8^6*s*^2^) and B_8_^2−^ at the PBE/TZP level. Similar diagrams for La/PrB_8_^−^ and Tm/YbB_8_^−^ are given in Supplementary Fig. 5. The dashed lines show the major contribution to the orbital interactions. The arrows on the 4 *f* orbitals represent *f*^8^ occupied electrons. The occupied 4*f* bands in TbB_8_^−^ are indicated by the slash solid lines. **b** The local coordinate system (LCS) analysis for the *C*_*7v*_ B_8_^2−^ ligand at the PBE/DZP level. The 32 valence orbitals of 2 *s*/2*p* characters are categorized into four groups. Herein, “t” and “r” represent “tangential” and “radial”, respectively. The subscript number corresponds to the nodal plane of the orbital contour. The superscript * indicates antibonding orbitals between the central B atom and the peripheral delocalized orbitals.

The *C*_*7v*_ structure of TbB_8_^−^ gives rise to a calculated VDE_1_/ADE at 2.05/1.93 eV at the PBE0/TZP level (Supplementary Table 7), in good agreement with the experimental value from band X at 1.98/1.87 eV. Peaks X and A both correspond to electron detachment from the 4*a*_1_ (Tb 6*s*) doubly occupied orbital (Supplementary Table 3, Fig. 3a and Supplementary Fig. 8) with different spin states. Peak B primarily represents detachment from the 2*e*_1_ bonding MO between the Tb 5*d*_*π*_ and B_8_ *π* orbitals (Supplementary Fig. 8). Bands C and D correspond to the 1*e*_1_ and 1*e*_3_ orbitals, respectively, primarily of in-plane B–B bonding characters. As shown previously^41,44,46^, the detachment cross-sections of 4*f*-based MOs are very weak and they are usually buried in the strong detachment transitions from the boron-based MOs, which is why Ln-doped boron clusters with the same structures usually give rise to similar photoelectron spectra, despite their different 4 *f* electron configurations. Overall, the good agreement between the experimental and theoretical data confirms the *C*_*7v*_ structure as the global minimum of TbB_8_^−^.

The computed VDE_1_/ADE for the *C*_*7v*_ global minimum of TmB_8_^−^ are 1.93/1.83 eV at the CCSD(T)/Def2-TZVP level (Supplementary Table 7), which agree with the observed value at 2.02/1.90 eV. Since the detachment cross-sections for *f*-based MOs are known to be low^41,44,46–49^, Supplementary Table 4 shows that band A should predominantly correspond to electron detachment from the 2*e*_1_ bonding MO between Tm and B_8_ (Supplementary Fig. 9). Band B corresponds to detachments from the 1*e*_1_ and 1*e*_3_ orbitals (Supplementary Table 4). Feature C at the higher binding energy side should be due to detachment from the 2*a*_1_ and 1*a*_1_ orbitals, which are delocalized *σ* MOs over the B_8_ plane (Supplementary Fig. 9). The good agreement between the experimental and theoretical results (Fig. 1d) confirms unequivocally that the half-sandwich *C*_*7v*_ structure is the global minimum for TmB_8_^−^. The photoelectron spectrum of YbB_8_^−^ is almost identical to that of TmB_8_^−^. The calculated detachment transitions for the *C*_*7v*_ global minimum for YbB_8_^−^ are also in excellent agreement with the experimental data, as shown in Fig. 1e and Supplementary Table 5.

### Unexpected structural and OS variations for the early and late lanthanide octa-boron clusters

The LnB_8_^−^ series of lanthanide octa-boron clusters were expected to exhibit similar structures and photoelectron spectra, as was the case observed previously for the di-lanthanide Ln_2_B_8_^−^ inverse sandwich complexes for Ln = La, Pr, Tb^44^. Surprisingly, we observed very different photoelectron spectra for the LnB_8_^−^ clusters, from the complicated spectra for the earlier lanthanides to the simpler spectral features in the late lanthanides. These experimental observations were borne out by the structural variations from our theoretical studies. As discussed above, the global minima of LaB_8_^−^ and PrB_8_^−^ were found to be 3D structures with *C*_*s*_ symmetry. A high symmetry *C*_*7v*_ structure was shown to be a low-lying isomer present experimentally along with the *C*_*s*_ global minima for both systems. We further found that the Ln atoms in the two structures adopt different OS:+III for the *C*_*s*_ isomer (4 *f*^*n*−3^) and +I for the *C*_*7v*_ isomer (5*d*^1^6 *s*^1^/4*f*^3^6 *s*^1^ for La/Pr). The early lanthanides tend to lose more electrons to form higher oxidation states because their 4*f*/5*d* orbitals are less contracted and closer to each other in energy (Supplementary Fig. 5). Because of the preference of Ln(III) OS for these early lanthanides, their empty 5*d* orbitals tend to bond stronger with the B_*n*_ ligands, so that the *C*_*7v*_ B©B_7_ wheel is distorted to the low-symmetry *C*_*s*_ B©B_6+1_ ligand to facilitate stronger Ln–B interactions.

The late lanthanides prefer to form lower OS due to the more contracted *f* orbitals (Fig. 3)^47,48^. Even though the *C*_*7v*_ structure is the global minimum for the middle-lanthanide Tb, the spectrum of TbB_8_^−^ is different from those of the late lanthanides TmB_8_^−^ and YbB_8_^−^, because of the different electronic configurations of the 6*s* orbital. As can be seen in Supplementary Table 8, the 6*s* orbital prefers to be singly occupied for all the *C*_*7v*_ LnB_8_^−^ species except for TbB_8_^−^, for which the 6*s* orbital is doubly occupied. The TDDFT-PBE results showed that the state with the 4*f*^9^6*s*^1^ configuration is 0.44 eV higher in energy than that for 4*f*^8^6 *s*^2^ (Supplementary Table 8). In TbB_8_^−^, the 6*s*-based MO (4*a*_1_ in Supplementary Fig. 8) also shows a significant contribution (~8%) from the center B atom of the B_8_ ligand, while this contribution is negligible (~2%) in all other *C*_*7v*_ LnB_8_^−^ species. Hence, the 6*s* orbital is slightly more stabilized by the high-lying ligand orbitals in TbB_8_^−^, resulting in its full occupation (Fig. 3). In view of the likely configuration mixing in this species, ab initio multiconfigurational calculations were carried out with complete-active-space self-consistent field (CASSCF) and the results are shown in Supplementary Fig. 11. It was found that the 4 *f*^8^6*s*^2^ configuration was slightly mixed with 4 *f*^8^5*d*^2^ (12%), but the OS should not be affected by the small multiconfigurational character. The OS change from early to late lanthanides can be explained qualitatively by the reduction of the lanthanide atomic sizes due to the 4*f* orbital contractions. Overall, the structure transition of the LnB_8_^−^ series from *C*_*s*_ to *C*_*7v*_ can be understood by the preferred OS due to the orbital energies and radial contractions of the 4*f*/5*d* orbitals.

Supplementary Table 9 presents the energy decomposition analysis (EDA)^50^ for all the LnB_8_^−^ species with their relative total energies decomposed into different terms to understand the relative stabilities of the *C*_*7v*_ and the *C*_*s*_ isomers. The energetic competition between the steric effect *(*Δ*E*_steric_, the sum of Pauli repulsion and electrostatic effect) and orbital interaction (Δ*E*_orb_) is the key to determining the overall stability of the clusters. In the early LaB_8_^−^ and PrB_8_^−^ species, the stabilization of Δ*E*_orb_ in the *C*_*7v*_ isomer is less than the stabilization of Δ*E*_steric_ in the *C*_*s*_ isomer, due to the elimination of Pauli repulsion between the Ln 6*s*^1^ and the ligand-based electrons in Ln(I). so that the *C*_*7v*_ isomer is higher in total energy than the *C*_*s*_ isomer. However, the opposite is true for TbB_8_^−^, TmB_8_^−^, and YbB_8_^−^, for which the *C*_*7v*_ structure shows stronger orbital interactions with the increased lanthanide contraction.

### Chemical bonding analyses

The *C*_*7v*_ structure can be viewed as a monovalent Ln(I) interacting with a doubly aromatic B_8_^2−^ ligand. Neutral B_8_ was known to be a triplet with two unpaired electrons with *D*_*7h*_ symmetry^36^. The closed-shell B_8_^2−^ was realized in the LiB_8_^−^ cluster due to charge transfer from Li to the B_8_ moiety^51^. To understand the chemical bonding in the *C*_*7v*_ LnB_8_^−^, we carried out MO analyses as shown in Fig. 3 and Supplementary Fig. 5, illustrating the orbital correlations of LnB_8_^−^ with those of the Ln^+^ and B_8_^2−^ moieties. The MO pictures for the LnB_8_^−^ complexes are depicted in Supplementary Figs. 6 to 10. As shown in Fig. 3 and Supplementary Fig. 5, the 2*e*_1_ orbitals describe the main bonding interactions between Ln^+^ and B_8_^2−^, which is further verified by EDA in conjunction with the natural orbitals for chemical valence (NOCV)^50^ method (Supplementary Table 11). Electron detachment from the 2*e*_1_ orbital can be approximately characterized by the second main peak of the *C*_*7v*_ global minima in the photoelectron spectra (Fig. 1): peak B for TbB_8_^−^, and peak A for TmB_8_^−^ and YbB_8_^−^. The compositions of the 2*e*_1_ bonding orbital given in Supplementary Table 10, as well as the percentage of the electrostatic effect given in Supplementary Table 12, show consistently that ionic characters tend to be stronger for the late lanthanide complexes, as compared with the ionic KB_8_^−^ species. From the EDA-NOCV analysis presented in Supplementary Table 11, we also found a strong 6*s* deformation corresponding to ΔE_orb(1)_, due to slight mixing of the 5*d* orbitals and symmetry-adapted B_8_ group orbitals. The 4*f* orbitals are well known to be radially too contracted in the lanthanide elements to participate in chemical bonding. Due to the low oxidation state of Ln(+I) in LnB_8_^−^, the partially filled 4*f* shells remain almost atom-like with ferromagnetic character (Fig. 3a and Supplementary Fig. 5), giving rise to interesting magnetic properties with potential applications in single-molecule magnet^52–54^ and magnetic nanowire^55,56^.

**Fig. 4:**
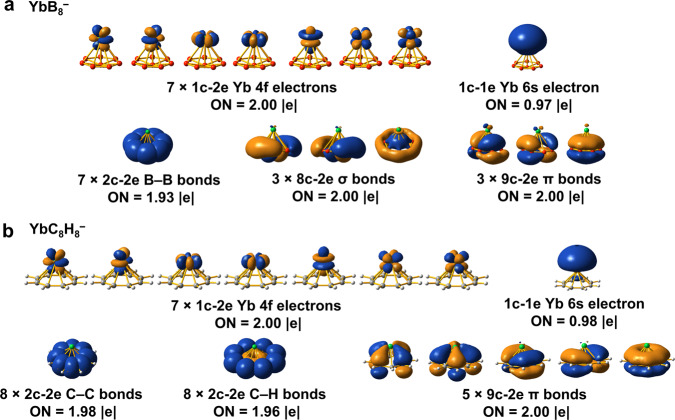
AdNDP bonding analyses of YbB_8_^−^ and comparison with that of YbC_8_H_8_^−^. **a** YbB_8_^−^ (*C*_*7v*_, ^2^A_1_). **b** YbC_8_H_8_^−^ (*C*_*8v*_, ^2^A_1_). The occupation numbers (ONs) are indicated.

Chemical bonding patterns obtained from the adaptive natural density partitioning (AdNDP) analyses^57^ can achieve a seamless description of different types of chemical bonds, recovering both Lewis-type bonding [one-center two-electron (1c-2e) lone pairs and classical two-center two-electron (2c-2e) bonds] and delocalized multicenter bonding associated with the concepts of aromaticity and antiaromaticity. Bonding schemes obtained from the AdNDP method for all the *C*_*7v*_ LnB_8_^−^ complexes are similar; the only differences are in the localized electrons in the Ln-based atomic-like orbitals. Figure 4a displays the AdNDP results of YbB_8_^−^, which has a closed 4*f*^*14*^ shell, to represent the bonding in all the *C*_*7v*_ LnB_8_^−^ complexes. The first row displays the seven pairs of the 4*f* electrons and the single unpaired 6*s* electron of Yb. The seven 2c-2e localized B–B bonds in the periphery of the B_8_^2−^ ligand are shown in the second row. Of particular importance are the two sets of multicenter bonds: the three delocalized in-plane 8c-2e *σ* bonds and the three delocalized 9c-2e *π* bonds. The latter represents *π* bonding interactions between the Yb 5*d* orbitals and the B_8_^2−^ ligand. The delocalized *σ* and *π* bonds of B_8_^2−^ are similar to those in B_7_^3−^ and B_9_^−^, giving rise to double aromaticity^36,41,51^. The AdNDP results for the *C*_*7v*_ and *C*_*s*_ isomers of LaB_8_^−^ are compared in Supplementary Fig. 12, showing that the two nonbonding unpaired La 6*s* and 5*d*_δ_ electrons in the *C*_*7v*_ isomer evolve into a 9c-2e *π* bond in the *C*_*s*_ isomer. The transformation of the two nonbonding La-based electrons in the *C*_*7v*_ isomer into a bonding pair in the *C*_*s*_ isomer explains why the latter is more stable, as well as why La exhibits +III OS in the *C*_*s*_ isomer.

The doubly aromatic B_8_^2−^ ligand is found to be analogous to the C_8_H_8_^2−^ aromatic cyclooctatetraenyl anion in terms of their planar structures and aromatic properties. The half-sandwich YbC_8_H_8_ complex was a well-known organometallic compound^58–61^, where Yb adopts +II OS. There are strong similarities in the chemical bonding between the monovalent YbC_8_H_8_^−^ and YbB_8_^−^, as shown in Fig. 4. The first row for both species is identical with seven 4*f* lone pairs and one unpaired 6*s* electron, suggesting a monovalent Yb(I). Similar to the 2c-2e B–B *σ* bonds in YbB_8_^−^, there are eight localized 2c-2e C–C *σ* bonds and eight 2c-2e C–H *σ* bonds in the second row on the C_8_H_8_^2−^ ligand. The five delocalized 9c-2e *π* bonds involve C 2*p*_π_ and 5*d* interactions, corresponding to the five delocalized *π* bonds of C_8_H_8_^2−^. Even though YbB_8_^−^ only has three delocalized aromatic *π* bonds, its *σ* aromaticity gives rise to additional stability.

### Boron cluster analogues of benzene (“borozene”)

**Fig. 5:**
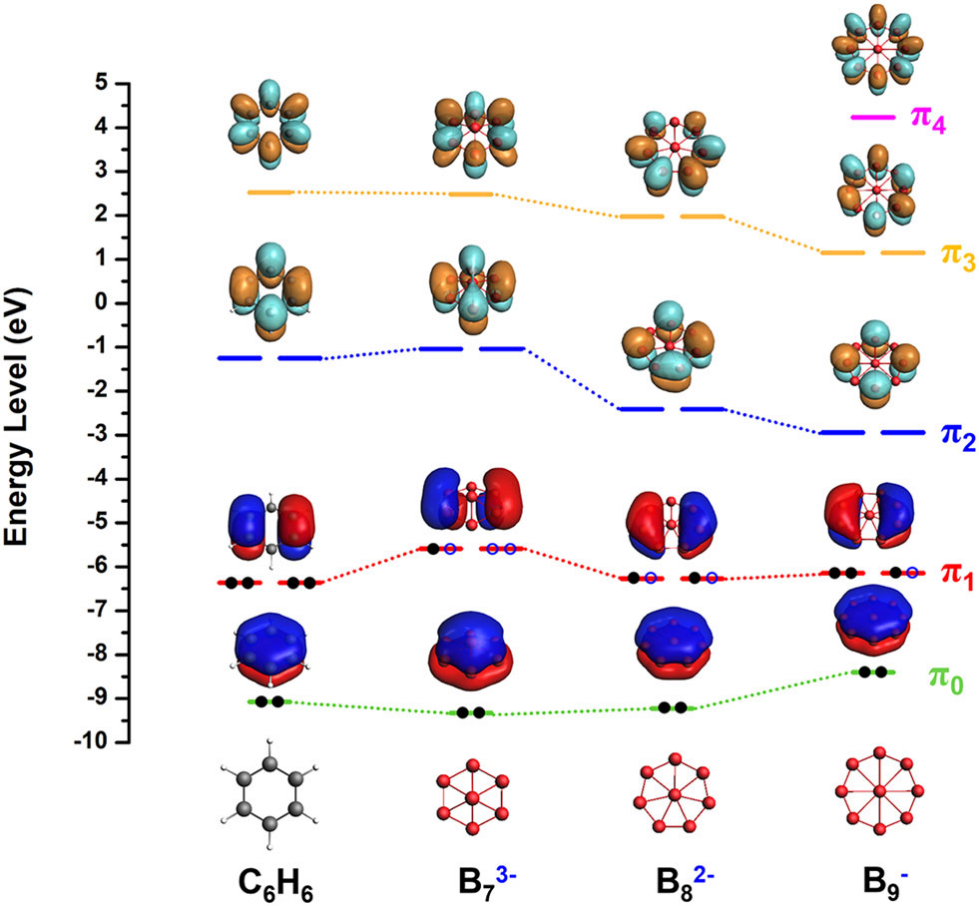
Comparison of the *π* orbitals of C_6_H_6_ with those of borozenes, B_7_^3−^, B_8_^2−^, and B_9_^−^ with different nodal planes. Black solid dots correspond to occupied electrons for the neutral species and blue circles represent the additional electrons in the closed-shell anions in the borozenes.

Most planar boron clusters are aromatic and their *π* electron systems are analogous to benzene or polycyclic aromatic hydrocarbons^29–36^. The planar B_7_^3−^, B_8_^2−^, and B_9_^−^ series are interesting; their *π* orbitals are compared with those of benzene in Fig. 5. Even though all these three boron clusters are also *σ* aromatic with six delocalized *σ* electrons, their *π* orbitals are almost identical to those of benzene. In fact, the trends of size and charge states of B_7_^3−^, B_8_^2−^, and B_9_^−^ are analogous to the C_5_H_5_^−^, C_6_H_6_, and C_7_H_7_^+^ series of aromatic hydrocarbons, respectively. Thus, this series of benzene-like aromatic boron clusters may be properly named as “borozene”. In fact, large planar aromatic boron clusters^30–33,35^ may be called “polycyclic aromatic borozenes” (PABs), analogous to polycyclic aromatic hydrocarbons (PAHs)^62^. We note that “borozene” was previously used for the planar B_12_H_6_ cluster, which was studied computationally^63^. However, the planar B_12_H_6_ structure was later found to be a very high energy isomer on the potential energy surface, where a partially hydrogenated 3D icosahedral-like B_12_H_6_ structure was found to be 35 kcal/mol lower in energy^64^. Thus, we think that “borozene” is more suitable for the B_7_^3−^, B_8_^2−^, and B_9_^−^ series of benzene-like aromatic planar boron clusters since the planar B_12_H_6_ species does not exist. Because the B_7_^3−^, B_8_^2−^, and B_9_^−^ borozenes are charged, they can be coordinated with lanthanide elements with tunable OSs. For example, the Pr atom is in +II OS in PrB_7_^−^, whereas in neutral PrB_7_ it is in +III OS^41^. It is conceivable that zero OS lanthanides may exist for late lanthanide LnB_9_^−^ clusters, similar to Ln(C_6_H_6_) complexes^65–67^. Supplementary Table 13 summarizes LnB_*n*_^−^ (*n* = 7–9) lanthanide borozene complexes with different OS of the lanthanides. Finally, the low-lying *π*_2_-MOs of borozenes (Fig. 5) are possible to accept four extra electrons to form sandwich-type compounds with actinides (An), similar to C_7_H_7_^3−^ in An(*η*^7^-C_7_H_7_)_2_ complexes^68^.

In conclusion, we report a joint photoelectron spectroscopy and quantum chemical study of lanthanide octa-boron clusters (LnB_8_^−^, Ln = La, Pr, Tb, Tm, Yb). For the early-lanthanide species (La and Pr), complicated photoelectron spectra are observed, whereas much simpler spectra are obtained for the late lanthanide species (Tb, Tm, Yb). The global minima of the early-lanthanide octa-boron clusters are found to be low-symmetry (*C*_*s*_) structures with a *C*_*7v*_ half-sandwich low-lying isomer that is also present experimentally, in agreement with the congested photoelectron spectra. The *C*_*7v*_ half-sandwich structure is found to be the global minimum for the late lanthanide (Tb, Tm, Yb) species, in accord to their relatively simple photoelectron spectral patterns. The *C*_*7v*_ half-sandwich octa-boron lanthanide complexes possess a rare monovalent Ln(I) center coordinated by a B_8_^2−^ ligand [Ln^I^(ƞ^8^-B_8_^2−^)]. The B_8_^2−^ ligand is doubly aromatic with six delocalized *π* and six delocalized *σ* electrons, underlying the stability of the monovalent Ln(I) complexes. The B_8_^2−^ ligand is a member of a class of doubly aromatic planar boron ligands (B_7_^3−^, B_8_^2−^, and B_9_^−^), named borozene. The current study represents a systematic characterization of monovalent lanthanide complexes coordinated with the B_8_^2−^ ligand, suggesting that borozenes with different charge states can serve as potential ligands to modulate oxidation states in lanthanide complexes.

## Methods

### Photoelectron spectroscopy

The experiments were performed using a magnetic-bottle photoelectron spectroscopy apparatus equipped with a laser vaporization supersonic cluster source, as shown schematically in Supplementary Fig. 1. More details for the apparatus could be found elsewhere^31,69^. In the current study, the Ln/^11^B target (5/2 mass ratio, Ln = La, Pr, Tb, Tm, Yb) was prepared by mixing a Ln powder (Alfa Aesar, −200 mesh, 99.9%) and ^11^B-enriched powder (Alfa Aesar, 96% ^11^B-enriched, −100 mesh, 99.9% metal basis) in a glove box. The mixed Ln/^11^B powder was then cold-pressed into a 12 mm diameter disk target, which was then transferred into the vacuum chamber for the generations of binary Ln–B clusters using a laser vaporization supersonic cluster source.

The clusters were generated by focusing a 532 nm laser beam from the second harmonic of a Nd:YAG laser onto the Ln/^11^B targets. The laser-induced plasma was quenched by a helium carrier gas seeded with 5% argon, initiating nucleation and cluster formation. Nascent clusters inside the nozzle were entrained in the carrier gas and underwent a supersonic expansion. After passing a skimmer, anionic clusters were extracted from the collimated cluster beam for time-of-flight (TOF) mass spectrometric analyses. The LnB_8_^−^ clusters of current interest were mass selected and decelerated before being photodetached by the 193 nm radiation (6.424 eV) from an ArF excimer laser or the 355 nm radiation (3.496 eV) from the third harmonic of a Nd:YAG laser. The photoelectron spectra were calibrated using the known spectrum of Bi^−^. The kinetic energy resolution of the apparatus was about 2.5%, i.e., 25 meV for 1 eV electrons.

### Theoretical methods

Because of the similarities of the observed photoelectron spectra and the anticipated similar structures, we performed more thorough global minimum searches only for LaB_8_^−^ and YbB_8_^−^ with different spin multiplicities using the TGMin 2.0 package^70–72^. More than 300 trial structures for each species were examined using the ADF 2017.114 software^73^ with the PBE density functional^74^ and the triple-ζ Slater-type plus one polarization function (TZP) basis set^75^. Herein, the frozen-core approximation was applied to the inner shells [1*s*^2^4*d*^10^] for lanthanides and [1*s*^2^] for B in the all-electron ADF calculations. The scalar relativistic effects were taken into consideration by the zero-order regular approximation^76^. Calculations using the hybrid PBE0 functional^77^ and TZP basis sets were further carried out to correct the relative energies of different isomers. Since two isomers were found to compete for the global minima of LaB_8_^−^ and PrB_8_^−^, single-point calculations at the CCSD(T) level were performed with the Def2-TZVP basis sets for the two lowest isomers, implemented in the ORCA software^78^. As low-valent metal compounds tend to possess multireference features, we checked these possibilities in our calculations. The T1 diagnostic factors in the CCSD calculations are 0.036, 0.039, 0.027, and 0.025 for LaB_8_^−^, PrB_8_^−^, TmB_8_^−^, and YbB_8_^−^, respectively, indicating the multiconfigurational characters were not significant as they lie within the accepted threshold of T1 < 0.04 for open-shell systems. Therefore, the DFT methods with single Slater determinant can well describe the ground states of these lanthanide species^41,44^. For TbB_8_^−^, however, a strong multireference character was found with higher T1. Consequently, we further determined the oxidation states using ab initio complete active space SCF method (CASSCF), where the active space included 14 electrons in 12 orbitals, consisting of seven 4 *f* orbitals, one 6 *s* orbital, two *d*-*p*_π_ bonding orbitals (mainly derived from B 2*p* orbitals) and two corresponding *d*-*p*_π_^*^ antibonding orbitals (mainly derived from Tb 5*d* orbitals). The ECP28MWB SDD pseudopotential and the SEG basis set was used for Tb^79–81^ and the cc-pVTZ basis set for B^82^.

Photoelectron spectra of LaB_8_^−^, PrB_8_^−^, TmB_8_^−^, and YbB_8_^−^ were simulated using the ΔSCF-TDDFT^83^ approach along with the SAOP model^84^. The first vertical detachment energy (VDE_1_) was computed as the difference in energy between the anionic ground state and the corresponding neutral at the same anionic geometry. The adiabatic detachment energy (ADE) was calculated as the energy difference between the anionic and neutral species at their respective optimized structures. We found the TDDFT method, used to compute higher VDEs, was not suitable to simulate the spectrum of TbB_8_^−^, probably due to the stronger spin contamination and the correlation effects. Thus, we used generalized Koopman’s theorem (GKT)^85^ based on Kohn-Sham orbitals to obtain the higher theoretical VDEs for the TbB_8_^−^ cluster. Chemical bonding analyses were performed using molecular orbital (MO) theory at the PBE0/TZP level and the AdNDP method^57^, where the first-order reduced density matrix was diagonalized with optimal convergence of the electron density description. At every step in the search for *n*c-2e bonds, the density matrix is depleted of the density, corresponding to the appropriate bonding elements and finally generating 1c-2e, 2c-2e, …, and *n*c-2e bonds. The Ln^+^…B_8_^2−^ interactions in the *C*_*7v*_ isomers were further analyzed with the EDA-NOCV method^50^ at the level of PBE/TZ2P.

## Supplementary information

Supplementary Information

## Data Availability

The data that support the findings of this study are available within the article and the associated Supplementary information. Any other data are available from the corresponding authors upon request.

## Code availability

The TGMin code used for the global minimum search is available from the corresponding author (J.L.) upon request and signing a license.
