# Supplementary material for: Monovalent lanthanide(I) in borozene complexes
Source: Nat Commun. 2021 Nov 9;12:6467. doi: 10.1038/s41467-021-26785-9 (PMC8578558; doi:10.1038/s41467-021-26785-9)
Supplement: Supplementary file 1 — Supplementary Information [file 41467_2021_26785_MOESM1_ESM.pdf]

Supplementary Information

Monovalent Lanthanide(I) in Borozene Complexes

Li, et al.

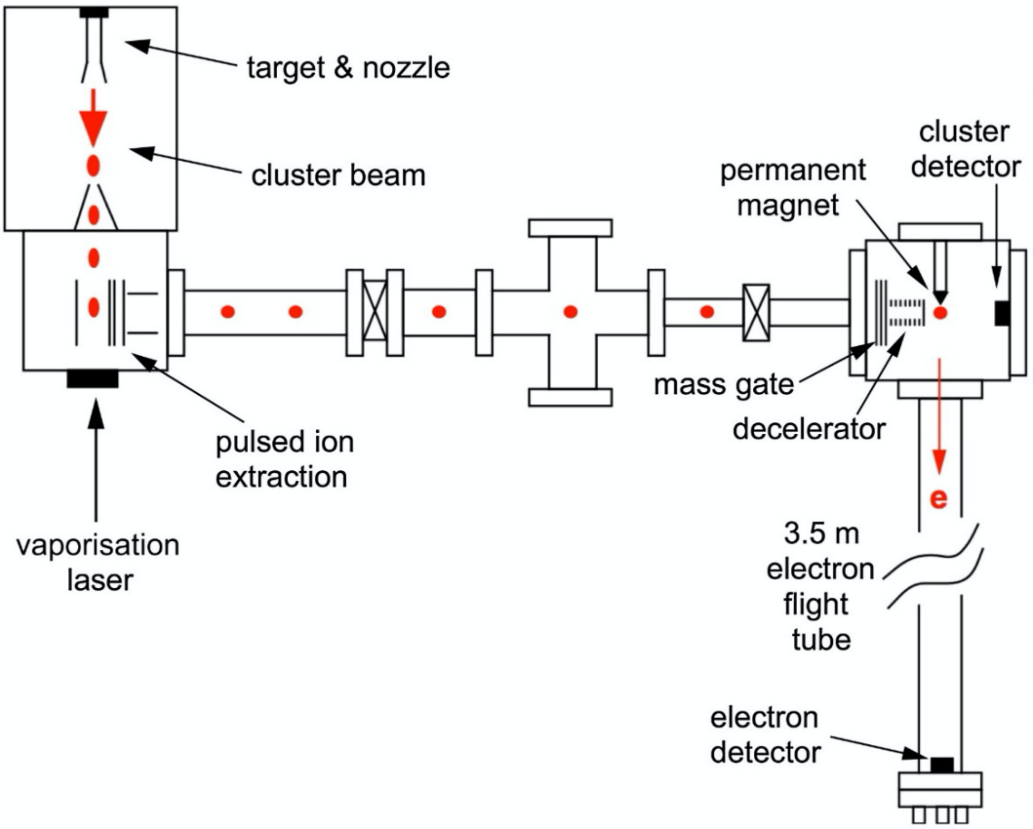

Supplementary Fig. 1 A schematic drawing of the magnetic-bottle PES apparatus equipped with a laser vaporization supersonic cluster source. Key components are labeled. The red dots represent the trajectory of the clusters.

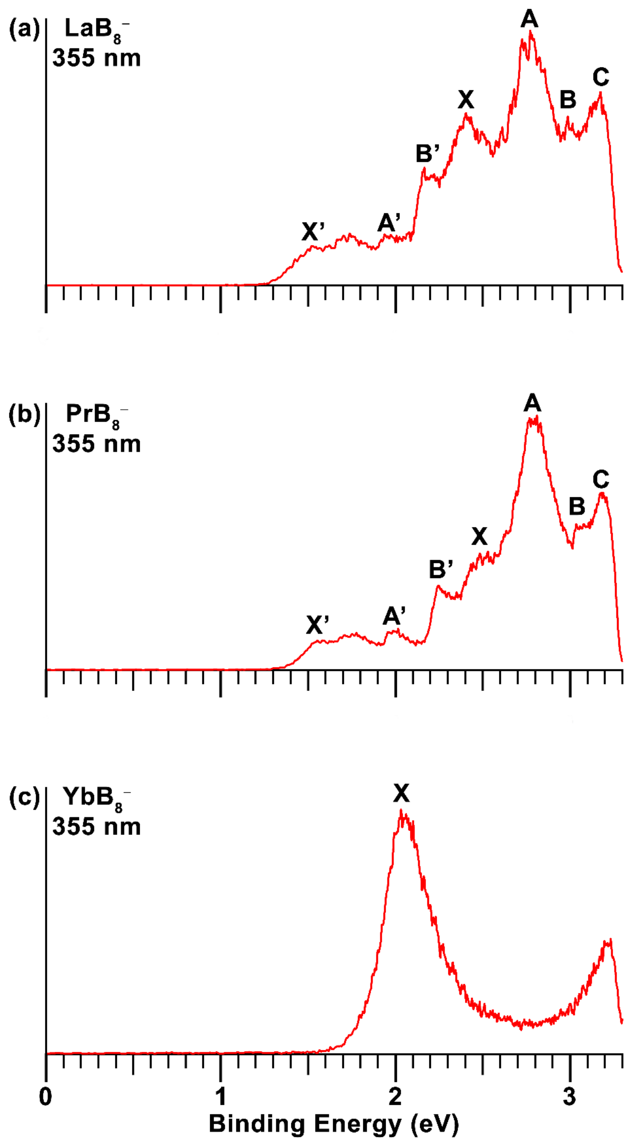

Supplementary Fig. 2 Photoelectron spectra of (a) LaB_8^-, (b) PrB_8^-, and (c) YbB_8^- at 355 nm (3.496 eV).

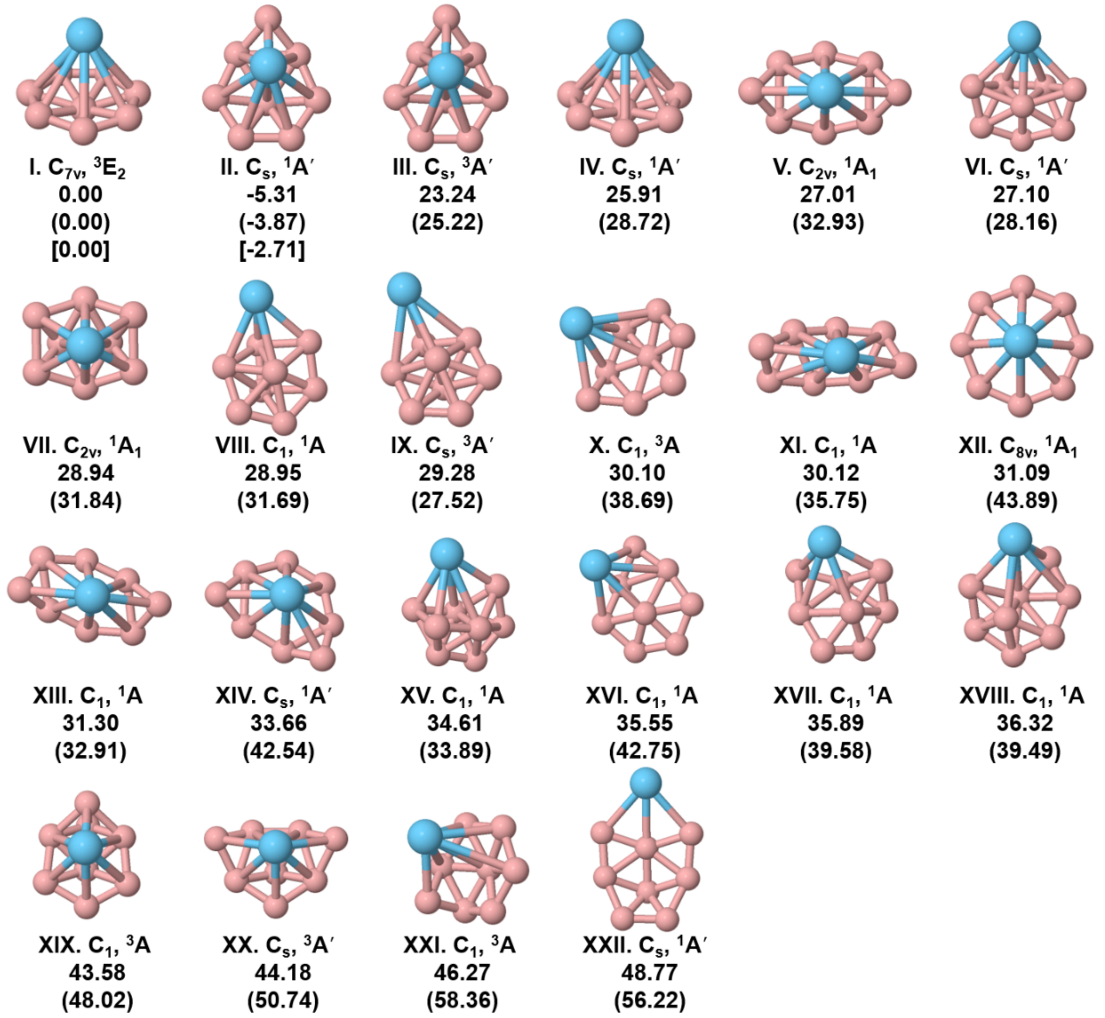

Supplementary Fig. 3 Optimized structures of low-lying isomers of LaB_8^- within 50 kcal/mol of the C_{7v} ($^3E_2$) structure at the levels of PBE/TZP and PBE0/TZP (in parenthesis).

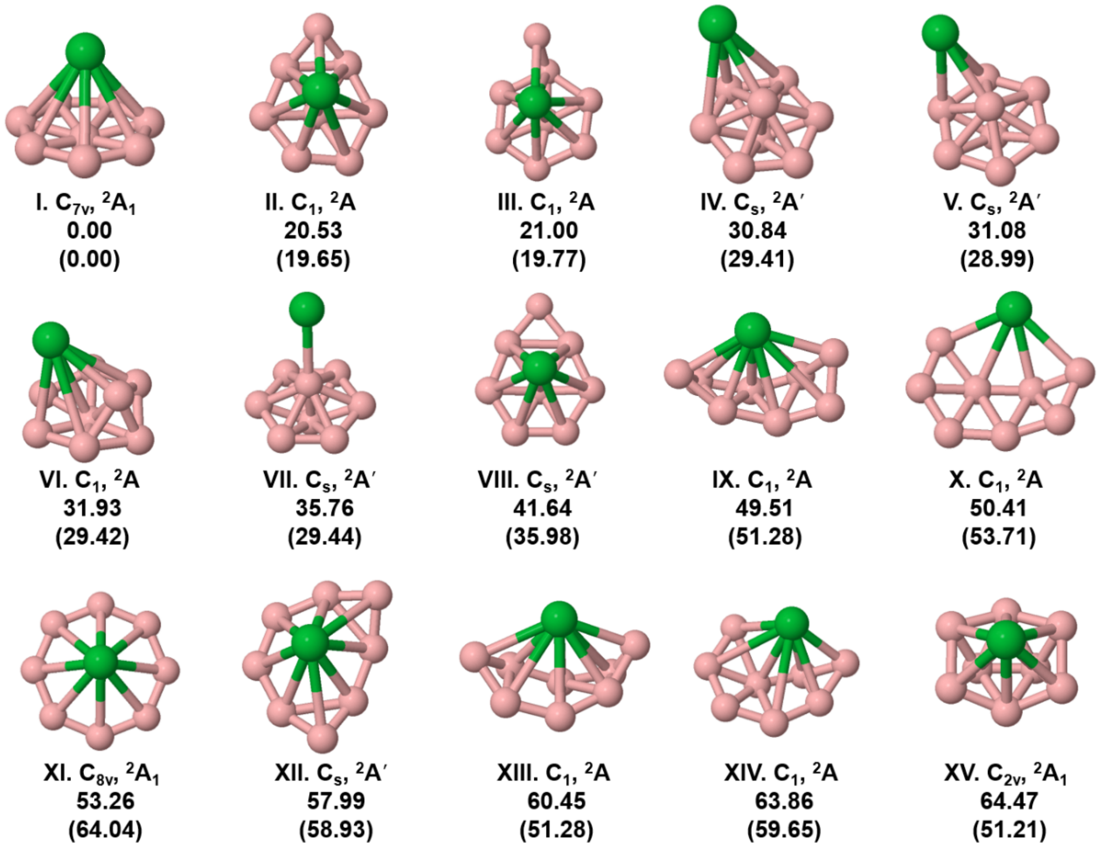

Supplementary Fig. 4 Optimized structures of low-lying isomers of YbB_8^- within 65 kcal/mol of the C_{7v} global minimum ($^2A_1$) at the levels of PBE/TZP and PBE0/TZP (in parenthesis).

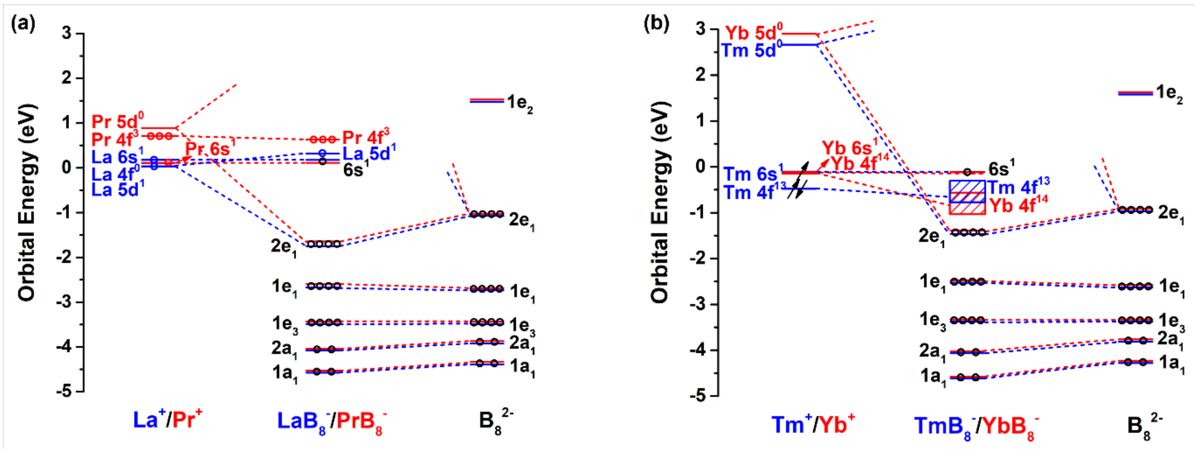

Supplementary Fig. 5 Orbital correlation diagrams of Ln^{+}, B_8^{2-} and LnB_8^{-}(C_{7v}) at the PBE/TZP level. (a) For the early lanthanides, La and Pr. (b) For the late lanthanide, Tm and Tb. The black circles correspond to the occupied electrons for different types of lanthanide-metal systems. The occupied 4*f* bands in TmB_8^{-} and YbB_8^{-} are labeled as slash solid lines.

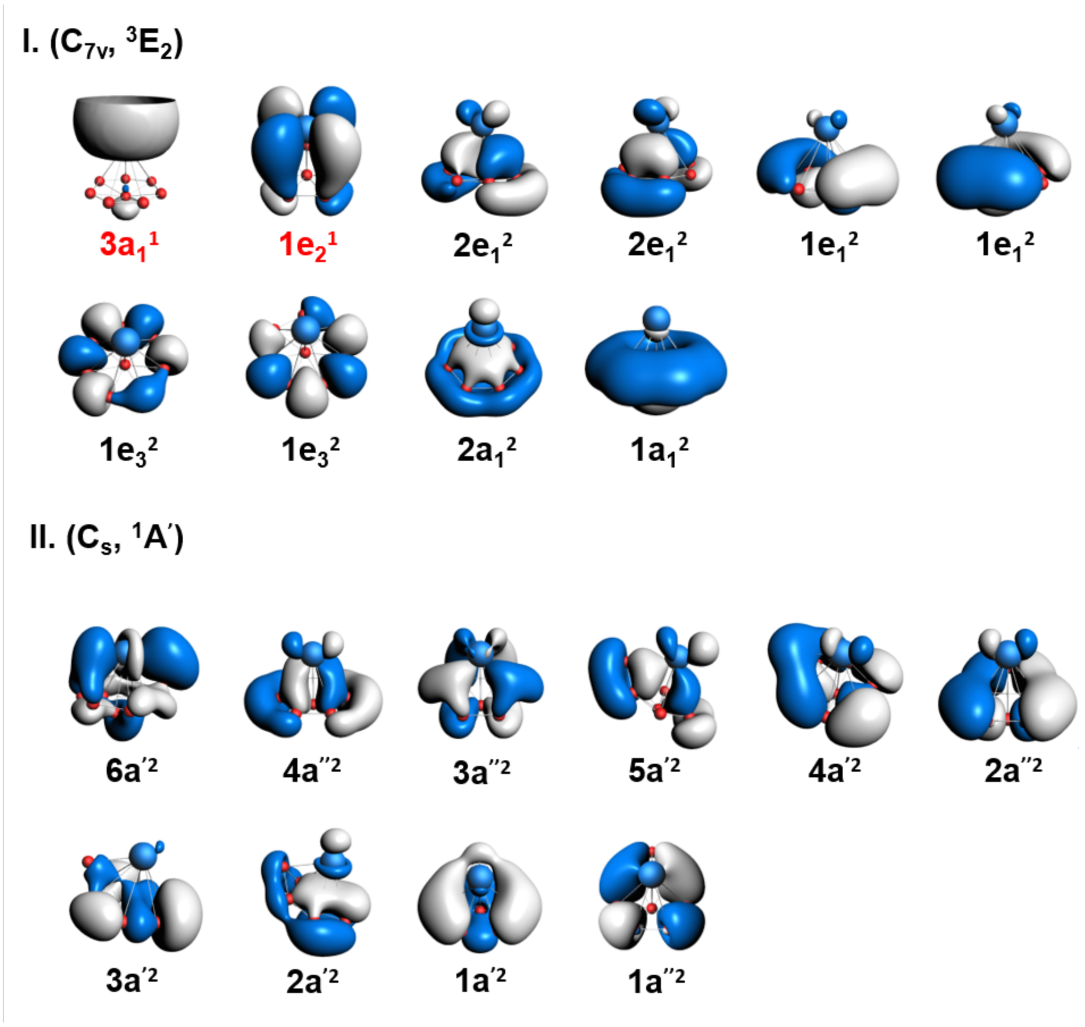

Supplementary Fig. 6 MO contours of isomer I (C_{7v}, $^3E_2$) and isomer II (C_s, $^1A'$) of LaB_8^{-} at the PBE/TZP level (isovalue = 0.03 au). Red labels correspond to singly occupied orbitals.

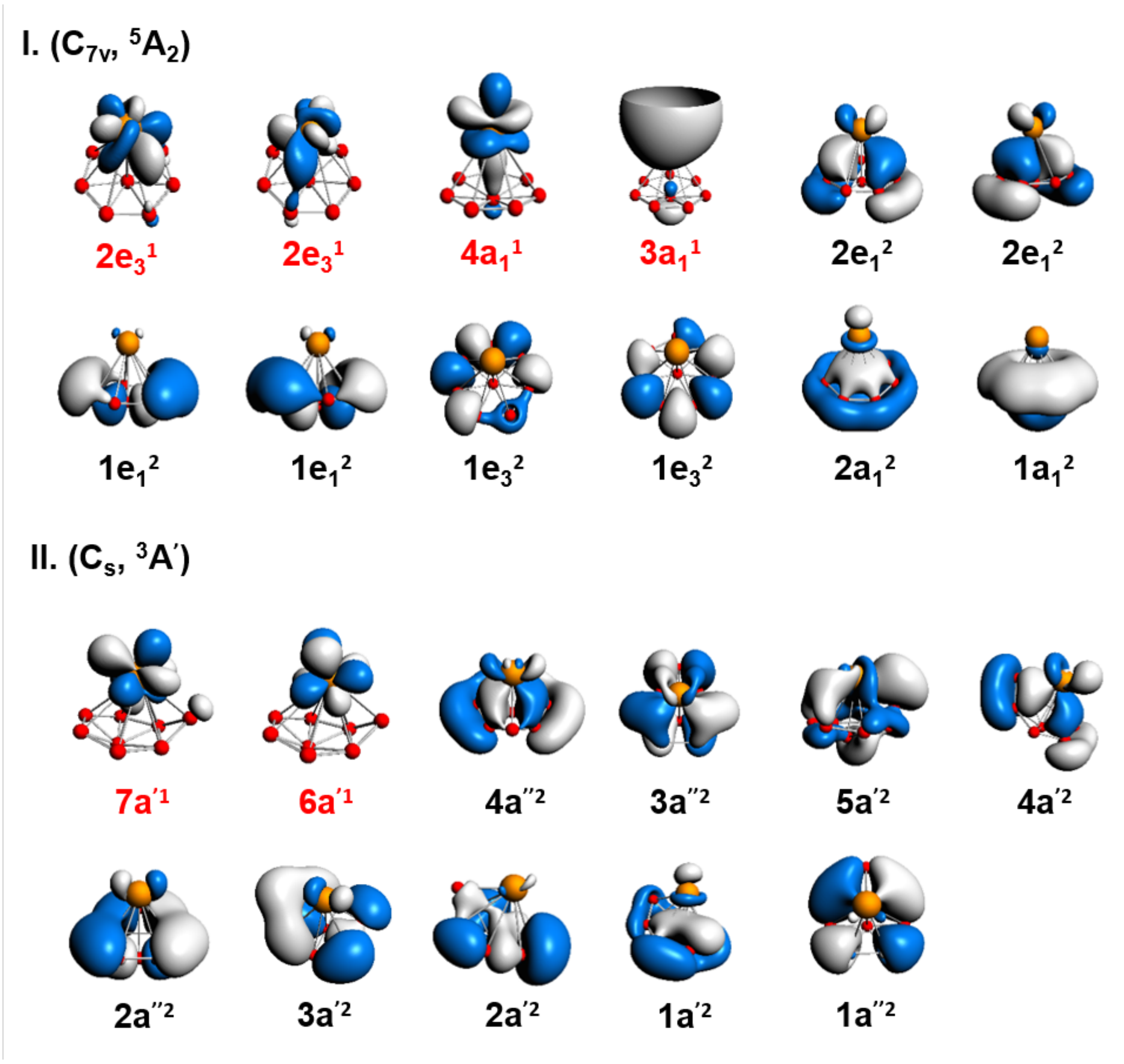

Supplementary Fig. 7 MO contours of isomer I (C_{7v}, $^{5}A_2$) and isomer II (C_s, $^{3}A'$) of PrB_8^- at the PBE/TZP level (isovalue = 0.03 au).

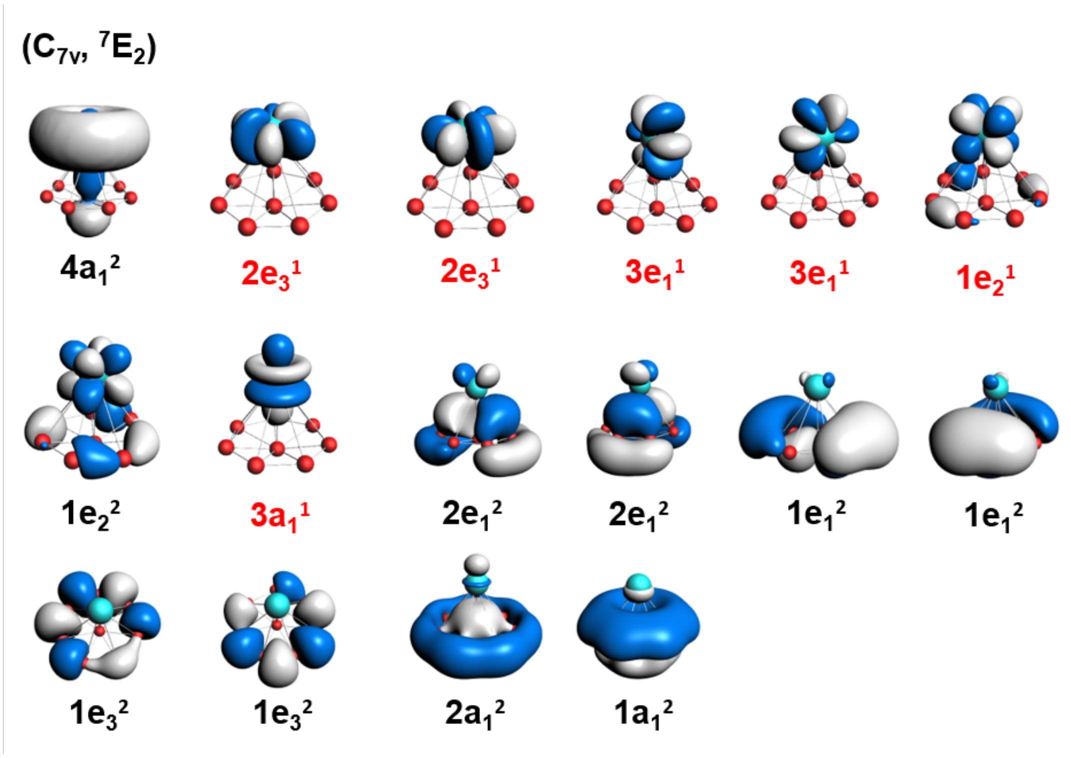

Supplementary Fig. 8 MO contours of isomer I (C_{7v}, $^7E_2$) of TbB_8^- at the PBE/TZP level (isovalue = 0.03 au).

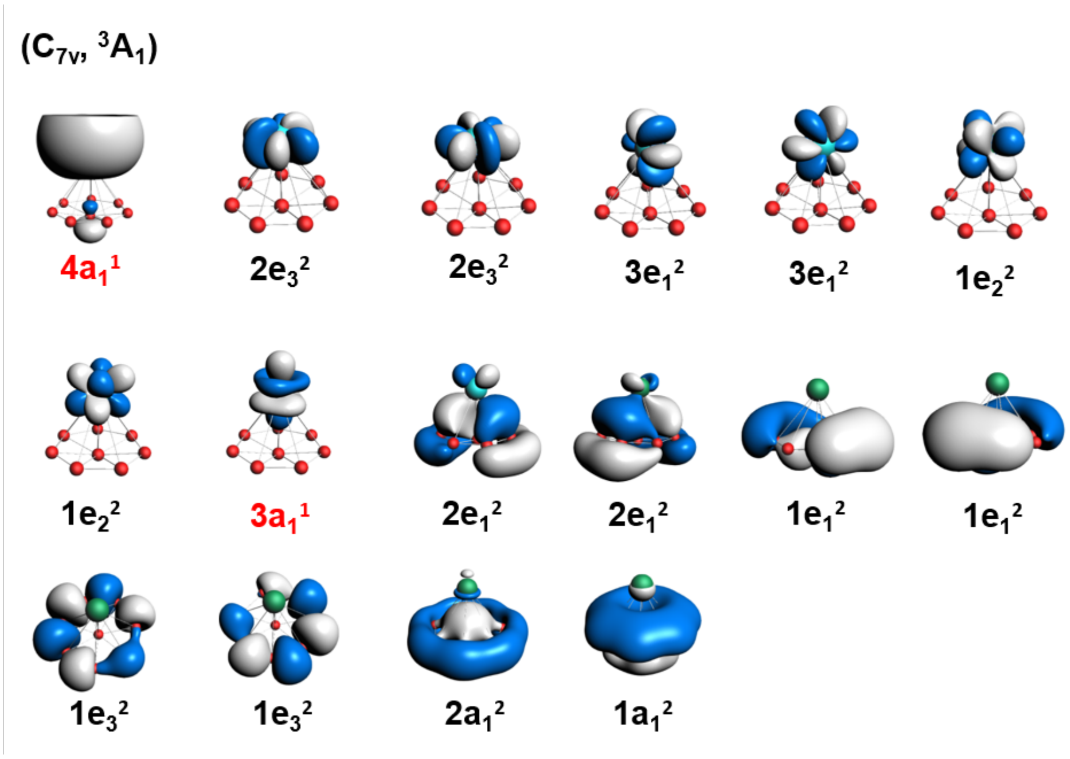

Supplementary Fig. 9 MO contours of isomer I (C_{7v}, $^3A_1$) of TmB_8^- at the PBE/TZP level (isovalue = 0.03 au).

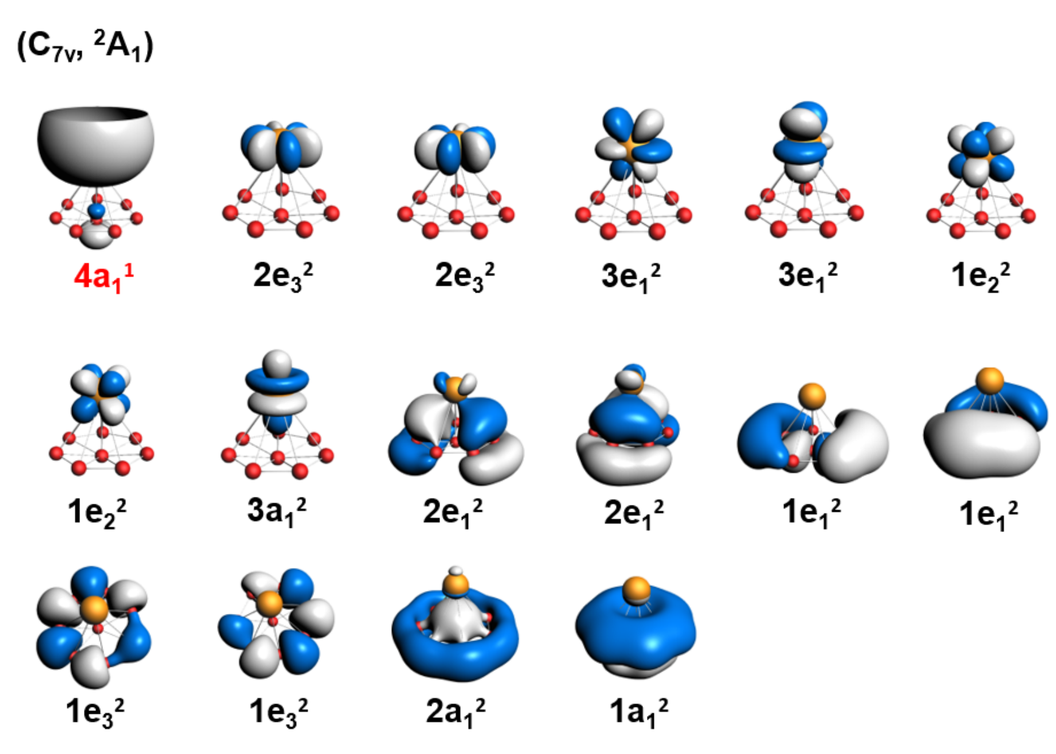

Supplementary Fig. 10 MO contours of isomer I (C_{7v}, $^2A_1$) of YbB_8^- at the PBE/TZP level (isovalue = 0.03 au).

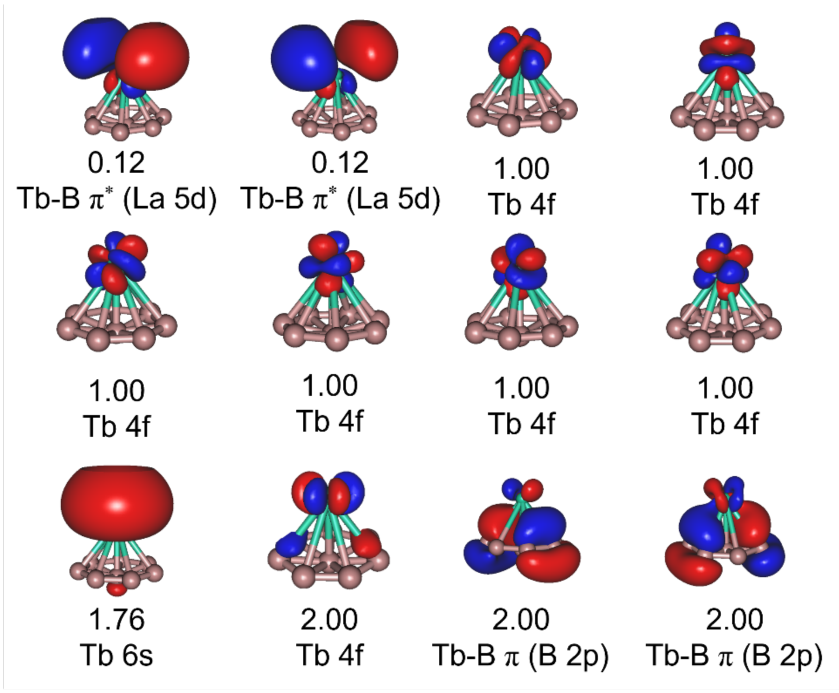

Supplementary Fig. 11 Natural valence orbital contours (cutoff = 0.03 au) of TbB_8^- (C_{7v}, $^7E_2$) from CASSCF (14e, 12o) at the PBE0/TZP optimized geometry. Values were natural occupation numbers with bonding characters. Highlights in parentheses are corresponding to the dominant contribution of the natural orbital. The CI coefficient of Tb(I) ($4f^86s^2$) is 88%, with 12% mixture of Tb(I) ($4f^85d^2$).

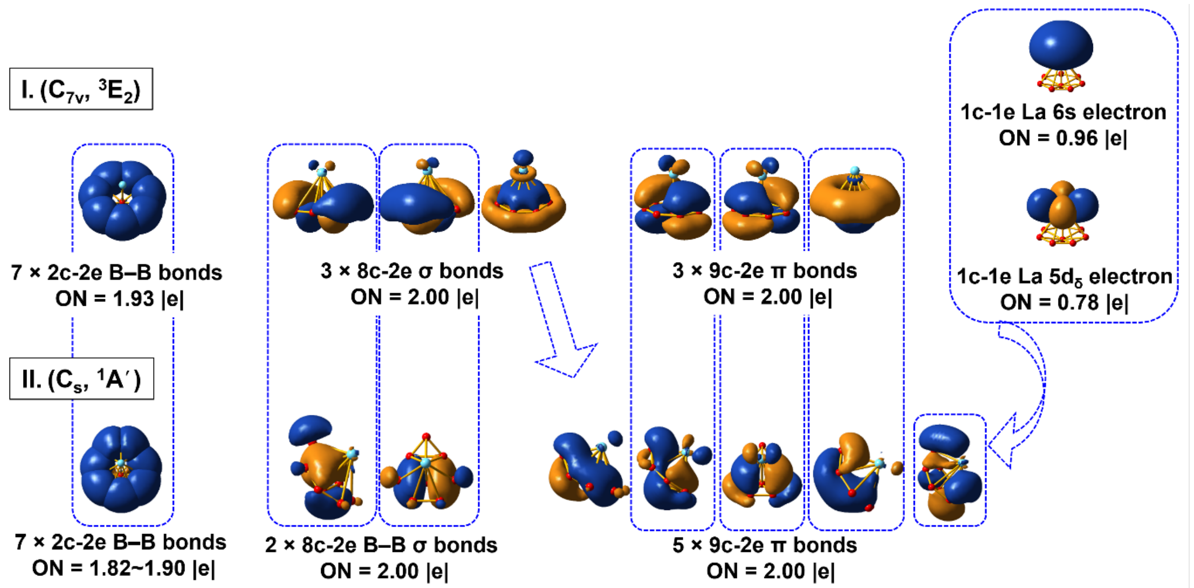

Supplementary Fig. 12 Results of AdNDP analyses for isomer I (C_{7v}, $^3E_2$) and isomer II (C_s, $^1A'$) of LaB_8^- at the PBE0/TZP level. Occupation numbers (ONs) are indicated. Bonding elements connected by the dash blocks or arrows are one-to-one correspondence in each isomer for comparison. Especially, the rightmost panel illustrates that the two 1c-1e bonds of La 6*s*/5*d* characters in isomer I evolve into a 9c-2e *π* bond in isomer II.

Supplementary Table 1. The measured vertical detachment energies (VDEs) for LaB_8^- in comparison with theoretical calculations for the C_s global minimum and the C_{7v} low-lying isomer. The bold-face indicates the orbitals from which an electron is detached. All energies are in eV.

Feature	Expt. VDE	Electronic Configuration	Theo. VDE	Final State
		C_{7v} LaB_8^-		
X'	~1.5	$\ldots 1a_1^2 2a_1^2 1e_3^4 1e_1^4 2e_1^4 \mathbf{3a_1^0} 1e_2^1$	1.54	$^2E_2$
A'	~1.9	$\ldots 1a_1^2 2a_1^2 1e_3^4 1e_1^4 2e_1^4 3a_1^1 \mathbf{1e_2^0}$	1.79	$^2A_1$
B'	2.16	$\ldots 1a_1^2 2a_1^2 1e_3^4 1e_1^4 \mathbf{2e_1^3} 3a_1^1 1e_2^1$	3.53	$^2E_1$ ($^2E_2$)[a]
		$\ldots 1a_1^2 2a_1^2 1e_3^4 1e_1^4 \mathbf{2e_1^3} 3a_1^1 1e_2^1$	3.59	$^4E_1$ ($^4E_2$)
		$\ldots 1a_1^2 2a_1^2 1e_3^4 \mathbf{1e_1^3} 2e_1^4 3a_1^1 1e_2^1$	4.48	$^2E_1$ ($^2E_2$)
		$\ldots 1a_1^2 2a_1^2 1e_3^4 \mathbf{1e_1^3} 2e_1^4 3a_1^1 1e_2^1$	4.57	$^4E_1$ ($^4E_2$)
		$\ldots 1a_1^2 2a_1^2 \mathbf{1e_3^3} 1e_1^4 2e_1^4 3a_1^1 1e_2^1$	5.39	$^2E_1$ ($^2E_2$)
		$\ldots 1a_1^2 2a_1^2 \mathbf{1e_3^3} 1e_1^4 2e_1^4 3a_1^1 1e_2^1$	5.49	$^4E_1$ ($^4E_2$)
		$\ldots 1a_1^2 \mathbf{2a_1^1} 1e_3^4 1e_1^4 2e_1^4 3a_1^1 1e_2^1$	5.87	$^2E_2$
		$\ldots 1a_1^2 \mathbf{2a_1^1} 1e_3^4 1e_1^4 2e_1^4 3a_1^1 1e_2^1$	6.00	$^4E_2$
		$\ldots \mathbf{1a_1^1} 2a_1^2 1e_3^4 1e_1^4 2e_1^4 3a_1^1 1e_2^1$	6.46	$^2E_2$
		$\ldots \mathbf{1a_1^1} 2a_1^2 1e_3^4 1e_1^4 2e_1^4 3a_1^1 1e_2^1$	6.59	$^4E_2$
		C_s LaB_8^-		
X	2.40	$\ldots 1a''^2 1a'^2 2a'^2 3a'^2 2a''^2 4a'^2 5a'^2 3a''^2 4a''^2 \mathbf{6a'^1}$	2.47	$^2A'$
A	2.77	$\ldots 1a''^2 1a'^2 2a'^2 3a'^2 2a''^2 4a'^2 5a'^2 3a''^2 \mathbf{4a''^1} 6a'^2$	2.74	$^2A''$
B	2.99	$\ldots 1a''^2 1a'^2 2a'^2 3a'^2 2a''^2 4a'^2 5a'^2 \mathbf{3a''^1} 4a''^2 6a'^2$	3.12	$^2A''$
C	3.18	$\ldots 1a''^2 1a'^2 2a'^2 3a'^2 2a''^2 4a'^2 \mathbf{5a'^1} 3a''^2 4a''^2 6a'^2$	3.23	$^2A'$
D	4.14	$\ldots 1a''^2 1a'^2 2a'^2 3a'^2 2a''^2 \mathbf{4a'^1} 5a'^2 3a''^2 4a''^2 6a'^2$	4.12	$^2A'$
E	4.70	$\ldots 1a''^2 1a'^2 2a'^2 3a'^2 \mathbf{2a''^1} 4a'^2 5a'^2 3a''^2 4a''^2 6a'^2$	4.57	$^2A''$
F	5.05	$\ldots 1a''^2 1a'^2 2a'^2 \mathbf{3a'^1} 2a''^2 4a'^2 5a'^2 3a''^2 4a''^2 6a'^2$	4.98	$^2A'$
G	~5.6	$\ldots 1a''^2 1a'^2 \mathbf{2a'^1} 3a'^2 2a''^2 4a'^2 5a'^2 3a''^2 4a''^2 6a'^2$	5.47	$^2A'$
H	~6.0	$\ldots 1a''^2 \mathbf{1a'^1} 2a'^2 3a'^2 2a''^2 4a'^2 5a'^2 3a''^2 4a''^2 6a'^2$	5.90	$^2A'$
		$\ldots \mathbf{1a''^1} 1a'^2 2a'^2 3a'^2 2a''^2 4a'^2 5a'^2 3a''^2 4a''^2 6a'^2$	6.78	$^2A''$

[a]States in parentheses are degenerate with the same excited energies. Final states are generated from the direct product of the irreducible representations under C_{7v} symmetry, as given in Supplementary Table 14.

Supplementary Table 2. The measured vertical detachment energies (VDEs) for PrB_8^- in comparison with theoretical calculations for the C_s global minimum and the C_{7v} low-lying isomer. The bold-face indicates the orbitals from which an electron is detached. All energies are in eV.

Feature	Expt. VDE	Electronic Configuration [a]	Theo. VDE	Final State
		C_{7v} PrB_8^-		
X'	~1.5	…$1a_1^2 2a_1^2 1e_3^4 1e_1^4 2e_1^4 \mathbf{3a_1^0} 4a_1^1 2e_3^2$	1.54	$^4A_2$
A'	~2.0	…$1a_1^2 2a_1^2 1e_3^4 1e_1^4 2e_1^4 3a_1^1 4a_1^1 \mathbf{2e_3^1}$	2.01	$^4E_3$
B'	2.25	…$1a_1^2 2a_1^2 1e_3^4 1e_1^4 2e_1^4 3a_1^1 \mathbf{4a_1^0} 2e_3^2$	2.33	$^4A_2$
		…$1a_1^2 2a_1^2 1e_3^4 1e_1^4 \mathbf{2e_1^3} 3a_1^1 4a_1^1 2e_3^2$	3.80	$^4E_1$
		…$1a_1^2 2a_1^2 1e_3^4 1e_1^4 \mathbf{2e_1^3} 3a_1^1 4a_1^1 2e_3^2$	3.84	$^6E_1$
		…$1a_1^2 2a_1^2 1e_3^4 \mathbf{1e_1^3} 2e_1^4 3a_1^1 4a_1^1 2e_3^2$	4.82	$^4E_1$
		…$1a_1^2 2a_1^2 1e_3^4 \mathbf{1e_1^3} 2e_1^4 3a_1^1 4a_1^1 2e_3^2$	4.94	$^6E_1$
		…$1a_1^2 2a_1^2 \mathbf{1e_3^3} 1e_1^4 2e_1^4 3a_1^1 4a_1^1 2e_3^2$	5.43	$^6E_3$
		…$1a_1^2 2a_1^2 \mathbf{1e_3^3} 1e_1^4 2e_1^4 3a_1^1 4a_1^1 2e_3^2$	5.45	$^4E_3$
		…$1a_1^2 \mathbf{2a_1^1} 1e_3^4 1e_1^4 2e_1^4 3a_1^1 4a_1^1 2e_3^2$	5.87	$^6A_2$
		…$1a_1^2 \mathbf{2a_1^1} 1e_3^4 1e_1^4 2e_1^4 3a_1^1 4a_1^1 2e_3^2$	6.00	$^4A_2$
		…$\mathbf{1a_1^1} 2a_1^2 1e_3^4 1e_1^4 2e_1^4 3a_1^1 4a_1^1 2e_3^2$	6.42	$^6A_2$
		…$\mathbf{1a_1^1} 2a_1^2 1e_3^4 1e_1^4 2e_1^4 3a_1^1 4a_1^1 2e_3^2$	6.55	$^4A_2$
		C_s PrB_8^-		
X	2.48	…$1a'^2 2a'^2 3a'^2 2a''^2 4a'^2 5a'^2 3a''^2 4a''^2 6a'^1 \mathbf{7a'^0}$	2.43	$^2A'$
A	2.81	…$1a'^2 2a'^2 3a'^2 2a''^2 4a'^2 5a'^2 3a''^2 \mathbf{4a''^1} 6a'^1 7a'^1$	2.80	$^4A''$
B	3.04	…$1a'^2 2a'^2 3a'^2 2a''^2 4a'^2 5a'^2 3a''^2 \mathbf{4a''^1} 6a'^1 7a'^1$	2.90	$^2A''$
C	3.18	…$1a'^2 2a'^2 3a'^2 2a''^2 4a'^2 \mathbf{5a'^1} 3a''^2 4a''^2 6a'^1 7a'^1$	3.31	$^4A'$
		…$1a'^2 2a'^2 3a'^2 2a''^2 4a'^2 5a'^2 \mathbf{3a''^1} 4a''^2 6a'^1 7a'^1$	3.37	$^2A''$
		…$1a'^2 2a'^2 3a'^2 2a''^2 4a'^2 5a'^2 \mathbf{3a''^1} 4a''^2 6a'^1 7a'^1$	3.44	$^4A''$
D	4.18	…$1a'^2 2a'^2 3a'^2 2a''^2 4a'^2 5a'^2 3a''^2 4a''^2 \mathbf{6a'^0} 7a'^1$	3.89	$^2A'$
		…$1a'^2 2a'^2 3a'^2 2a''^2 \mathbf{4a'^1} 5a'^2 3a''^2 4a''^2 6a'^1 7a'^1$	4.01	$^4A'$
		…$1a'^2 2a'^2 3a'^2 2a''^2 4a'^2 \mathbf{5a'^1} 3a''^2 4a''^2 6a'^1 7a'^1$	4.23	$^2A'$
		…$1a'^2 2a'^2 3a'^2 2a''^2 \mathbf{4a'^1} 5a'^2 3a''^2 4a''^2 6a'^1 7a'^1$	4.45	$^2A'$
E	4.85	…$1a'^2 2a'^2 \mathbf{3a'^1} 2a''^2 4a'^2 5a'^2 3a''^2 4a''^2 6a'^1 7a'^1$	4.81	$^2A'$
F	5.09	…$1a'^2 2a'^2 \mathbf{3a'^1} 2a''^2 4a'^2 5a'^2 3a''^2 4a''^2 6a'^1 7a'^1$	5.00	$^4A'$
		…$1a'^2 2a'^2 3a'^2 \mathbf{2a''^1} 4a'^2 5a'^2 3a''^2 4a''^2 6a'^1 7a'^1$	5.30	$^2A''$
		…$1a'^2 2a'^2 3a'^2 \mathbf{2a''^1} 4a'^2 5a'^2 3a''^2 4a''^2 6a'^1 7a'^1$	5.42	$^2A''$
G	~5.7	…$1a'^2 2a'^2 \mathbf{3a'^1} 2a''^2 4a'^2 5a'^2 3a''^2 4a''^2 6a'^1 7a'^1$	5.68	$^2A'$
		…$1a'^2 2a'^2 \mathbf{3a'^1} 2a''^2 4a'^2 5a'^2 3a''^2 4a''^2 6a'^1 7a'^1$	5.71	$^4A'$
		…$1a'^2 \mathbf{2a'^1} 3a'^2 2a''^2 4a'^2 5a'^2 3a''^2 4a''^2 6a'^1 7a'^1$	6.04	$^2A'$
H	~6.2	…$1a'^2 \mathbf{2a'^1} 3a'^2 2a''^2 4a'^2 5a'^2 3a''^2 4a''^2 6a'^1 7a'^1$	6.20	$^4A'$
		…$\mathbf{1a'^1} 2a'^2 3a'^2 2a''^2 4a'^2 5a'^2 3a''^2 4a''^2 6a'^1 7a'^1$	6.46	$^2A'$
		…$\mathbf{1a'^1} 2a'^2 3a'^2 2a''^2 4a'^2 5a'^2 3a''^2 4a''^2 6a'^1 7a'^1$	6.62	$^4A'$

[a] In the C_{7v} isomer, $4a_1$ and $2e_3$ correspond to the 4*f* orbitals; in the C_s isomer, $6a'$ and $7a'$ are the 4*f* orbitals.

Supplementary Table 3. The measured vertical detachment energies (VDEs) for TbB_8^- in comparison with theoretical calculations for the C_{7v} global minimum. The bold-face indicates the orbitals from which an electron is detached. All energies are in eV.

Feature	Expt. VDE	Electronic Configuration [a]	Theo. VDE	Final State
X	1.98	$\ldots 1a_1^2 2a_1^2 1e_3^4 1e_1^4 2e_1^4 3a_1^1 1e_2^3 3e_1^2 2e_3^2 \mathbf{4a_1^1}$	2.05	$^8E_2$
A	2.18	$\ldots 1a_1^2 2a_1^2 1e_3^4 1e_1^4 2e_1^4 3a_1^1 \mathbf{1e_2^2} 3e_1^2 2e_3^2 4a_1^2$	2.16	$^8A_2$
		$\ldots 1a_1^2 2a_1^2 1e_3^4 1e_1^4 2e_1^4 3a_1^1 1e_2^3 3e_1^2 2e_3^2 \mathbf{4a_1^1}$	2.37 [b]	$^6E_2$
B	4.02	$\ldots 1a_1^2 2a_1^2 1e_3^4 1e_1^4 \mathbf{2e_1^3} 3a_1^1 1e_2^3 3e_1^2 2e_3^2 4a_1^2$	4.14	$^8E_1$ ($^8E_3$)[c]
		$\ldots 1a_1^2 2a_1^2 1e_3^4 1e_1^4 2e_1^4 \mathbf{3a_1^0} 1e_2^3 3e_1^2 2e_3^2 4a_1^2$	4.70	$^6E_2$
C	5.06	$\ldots 1a_1^2 2a_1^2 1e_3^4 \mathbf{1e_1^3} 2e_1^4 3a_1^1 1e_2^3 3e_1^2 2e_3^2 4a_1^2$	5.16	$^8E_1$ ($^8E_3$)
		$\ldots 1a_1^2 2a_1^2 1e_3^4 1e_1^4 2e_1^4 3a_1^1 1e_2^3 \mathbf{3e_1^1} 2e_3^2 4a_1^2$	5.18	$^6E_1$ ($^6E_3$)
		$\ldots 1a_1^2 2a_1^2 1e_3^4 1e_1^4 2e_1^4 3a_1^1 1e_2^3 3e_1^2 \mathbf{2e_3^1} 4a_1^2$	5.40	$^6E_1$ ($^6E_2$)
D	6.1	$\ldots 1a_1^2 2a_1^2 \mathbf{1e_3^3} 1e_1^4 2e_1^4 3a_1^1 1e_2^3 3e_1^2 2e_3^2 4a_1^2$	6.04	$^8E_1$ ($^8E_2$)

[a] $3a_1$, $1e_2$, $3e_1$ and $2e_3$ correspond to the 4*f* orbitals.

[b] The VDE value of this state is obtained from spin-flip TD-SAOP/TZP method.

[c] States in parentheses are degenerated with the same excited energies. Final states are generated from direct product of irreducible representations of C_{7v} symmetry, as listed in Supplementary Table 14.

Supplementary Table 4. The measured vertical detachment energies (VDEs) for TmB_8^- in comparison with theoretical calculations for the C_{7v} global minimum. The bold-face indicates the orbitals from which an electron is detached. All energies are in eV.

Feature	Expt. VDE	Electronic Configuration [a]	Theo. VDE	Final State
X	2.02	$\ldots 1a_1^2 2a_1^2 1e_3^4 1e_1^4 2e_1^4 3a_1^1 1e_2^4 3e_1^4 2e_3^4 \mathbf{4a_1^0}$	1.93	$^2A_1$
A	3.54	$\ldots 1a_1^2 2a_1^2 1e_3^4 1e_1^4 2e_1^4 3a_1^1 1e_2^4 3e_1^4 \mathbf{2e_3^3} 4a_1^1$	3.09	$^4E_3$
		$\ldots 1a_1^2 2a_1^2 1e_3^4 1e_1^4 \mathbf{2e_1^3} 3a_1^1 1e_2^4 3e_1^4 2e_3^4 4a_1^1$	3.28	$^4E_1$
		$\ldots 1a_1^2 2a_1^2 1e_3^4 1e_1^4 2e_1^4 3a_1^1 \mathbf{1e_2^3} 3e_1^4 2e_3^4 4a_1^1$	3.33	$^4E_2$
		$\ldots 1a_1^2 2a_1^2 1e_3^4 1e_1^4 \mathbf{2e_1^3} 3a_1^1 1e_2^4 3e_1^4 2e_3^4 4a_1^1$	3.37	$^2E_1$
		$\ldots 1a_1^2 2a_1^2 1e_3^4 1e_1^4 2e_1^4 3a_1^1 1e_2^4 3e_1^4 \mathbf{2e_3^3} 4a_1^1$	3.54	$^2E_3$
B	4.79	$\ldots 1a_1^2 2a_1^2 1e_3^4 1e_1^4 2e_1^4 3a_1^1 1e_2^4 \mathbf{3e_1^3} 2e_3^4 4a_1^1$	4.13	$^4E_1$
		$\ldots 1a_1^2 2a_1^2 1e_3^4 1e_1^4 2e_1^4 3a_1^1 \mathbf{1e_2^3} 3e_1^4 2e_3^4 4a_1^1$	4.22	$^2E_2$
		$\ldots 1a_1^2 2a_1^2 1e_3^4 \mathbf{1e_1^3} 2e_1^4 3a_1^1 1e_2^4 3e_1^4 2e_3^4 4a_1^1$	4.50	$^4E_1$
		$\ldots 1a_1^2 2a_1^2 1e_3^4 \mathbf{1e_1^3} 2e_1^4 3a_1^1 1e_2^4 3e_1^4 2e_3^4 4a_1^1$	4.57	$^2E_1$
		$\ldots 1a_1^2 2a_1^2 1e_3^4 1e_1^4 2e_1^4 3a_1^1 1e_2^4 \mathbf{3e_1^3} 2e_3^4 4a_1^1$	4.99	$^2E_1$
		$\ldots 1a_1^2 2a_1^2 \mathbf{1e_3^3} 1e_1^4 2e_1^4 3a_1^1 1e_2^4 3e_1^4 2e_3^4 4a_1^1$	5.37	$^4E_3$
		$\ldots 1a_1^2 2a_1^2 \mathbf{1e_3^3} 1e_1^4 2e_1^4 3a_1^1 1e_2^4 3e_1^4 2e_3^4 4a_1^1$	5.43	$^2E_3$
		$\ldots 1a_1^2 2a_1^2 1e_3^4 1e_1^4 2e_1^4 \mathbf{3a_1^0} 1e_2^4 3e_1^4 2e_3^4 4a_1^1$	5.49	$^2A_1$
C	6.1	$\ldots 1a_1^2 \mathbf{2a_1^1} 1e_3^4 1e_1^4 2e_1^4 3a_1^1 1e_2^4 3e_1^4 2e_3^4 4a_1^1$	5.94	$^4A_1$
		$\ldots 1a_1^2 \mathbf{2a_1^1} 1e_3^4 1e_1^4 2e_1^4 3a_1^1 1e_2^4 3e_1^4 2e_3^4 4a_1^1$	6.05	$^2A_1$
		$\ldots \mathbf{1a_1^1} 2a_1^2 1e_3^4 1e_1^4 2e_1^4 3a_1^1 1e_2^4 3e_1^4 2e_3^4 4a_1^1$	6.56	$^4A_1$
		$\ldots \mathbf{1a_1^1} 2a_1^2 1e_3^4 1e_1^4 2e_1^4 3a_1^1 1e_2^4 3e_1^4 2e_3^4 4a_1^1$	6.70	$^2A_1$

[a] $3a_1$, $1e_2$, $3e_1$ and $2e_3$ correspond to the 4*f* orbitals.

Supplementary Table 5. The measured vertical detachment energies (VDEs) for YbB_8^- in comparison with theoretical calculations for the C_{7v} global minimum. The bold-face indicates the orbitals from which an electron is detached. All energies are in eV.

Feature	Expt. VDE	Electronic Configuration [a]	Theo. VDE	Final State
X	2.03	$\ldots 1a_1^2 2a_1^2 1e_3^4 1e_1^4 2e_1^4 3a_1^2 1e_2^4 3e_1^4 2e_3^4 \mathbf{4a_1^0}$	1.92	$^3A_1$
A	3.49	$\ldots 1a_1^2 2a_1^2 1e_3^4 1e_1^4 \mathbf{2e_1^3} 3a_1^2 1e_2^4 3e_1^4 2e_3^4 4a_1^1$	3.45	$^1E_1$
		$\ldots 1a_1^2 2a_1^2 1e_3^4 1e_1^4 \mathbf{2e_1^3} 3a_1^2 1e_2^4 3e_1^4 2e_3^4 4a_1^1$	3.49	$^3E_1$
A'	3.97	$\ldots 1a_1^2 2a_1^2 1e_3^4 1e_1^4 2e_1^4 3a_1^2 \mathbf{1e_2^3} 3e_1^4 2e_3^4 4a_1^1$	4.01	$^1E_2$
		$\ldots 1a_1^2 2a_1^2 1e_3^4 1e_1^4 2e_1^4 3a_1^2 1e_2^4 3e_1^4 \mathbf{2e_3^3} 4a_1^1$	4.01	$^1E_3$
		$\ldots 1a_1^2 2a_1^2 1e_3^4 1e_1^4 2e_1^4 3a_1^2 1e_2^4 \mathbf{3e_1^3} 2e_3^4 4a_1^1$	4.02	$^1E_1$
		$\ldots 1a_1^2 2a_1^2 1e_3^4 1e_1^4 2e_1^4 3a_1^2 1e_2^4 3e_1^4 \mathbf{2e_3^3} 4a_1^1$	4.05	$^3E_3$
		$\ldots 1a_1^2 2a_1^2 1e_3^4 1e_1^4 2e_1^4 3a_1^2 \mathbf{1e_2^3} 3e_1^4 2e_3^4 4a_1^1$	4.06	$^3E_2$
		$\ldots 1a_1^2 2a_1^2 1e_3^4 1e_1^4 2e_1^4 \mathbf{3a_1^1} 1e_2^4 3e_1^4 2e_3^4 4a_1^1$	4.06	$^3A_1$
		$\ldots 1a_1^2 2a_1^2 1e_3^4 1e_1^4 2e_1^4 3a_1^2 1e_2^4 \mathbf{3e_1^3} 2e_3^4 4a_1^1$	4.08	$^3E_1$
		$\ldots 1a_1^2 2a_1^2 1e_3^4 1e_1^4 2e_1^4 \mathbf{3a_1^1} 1e_2^4 3e_1^4 2e_3^4 4a_1^1$	4.09	$^1A_1$
B	4.79	$\ldots 1a_1^2 2a_1^2 1e_3^4 \mathbf{1e_1^3} 2e_1^4 3a_1^2 1e_2^4 3e_1^4 2e_3^4 4a_1^1$	4.58	$^1E_1$
		$\ldots 1a_1^2 2a_1^2 1e_3^4 \mathbf{1e_1^3} 2e_1^4 3a_1^2 1e_2^4 3e_1^4 2e_3^4 4a_1^1$	4.63	$^3E_1$
		$\ldots 1a_1^2 2a_1^2 \mathbf{1e_3^3} 1e_1^4 2e_1^4 3a_1^2 1e_2^4 3e_1^4 2e_3^4 4a_1^1$	5.28	$^1E_3$
		$\ldots 1a_1^2 2a_1^2 \mathbf{1e_3^3} 1e_1^4 2e_1^4 3a_1^2 1e_2^4 3e_1^4 2e_3^4 4a_1^1$	5.32	$^3E_3$
C	~6.2	$\ldots 1a_1^2 \mathbf{2a_1^1} 1e_3^4 1e_1^4 2e_1^4 3a_1^2 1e_2^4 3e_1^4 2e_3^4 4a_1^1$	5.94	$^1E_3$
		$\ldots 1a_1^2 \mathbf{2a_1^1} 1e_3^4 1e_1^4 2e_1^4 3a_1^2 1e_2^4 3e_1^4 2e_3^4 4a_1^1$	5.98	$^3E_3$
		$\ldots \mathbf{1a_1^1} 2a_1^2 1e_3^4 1e_1^4 2e_1^4 3a_1^2 1e_2^4 3e_1^4 2e_3^4 4a_1^1$	6.46	$^1A_1$
		$\ldots \mathbf{1a_1^1} 2a_1^2 1e_3^4 1e_1^4 2e_1^4 3a_1^2 1e_2^4 3e_1^4 2e_3^4 4a_1^1$	6.86	$^3A_1$

[a] $3a_1$, $1e_2$, $3e_1$ and $2e_3$ correspond to the 4*f* orbitals.

Supplementary Table 6. The cartesian coordinates of the optimized structures of LnB_8^- (Ln = La, Pr, Tb, Tm, Yb) at the PBE0/TZP level. The structure of the optimized neutral LaB_8 was obtained using the PBE/TZP method due to challenges of geometrical convergence at the PBE0 level with accurate electron state.

LaB_8^-

I. (C_{7v}, $^3E_2$)

1.B	0.397717	1.742510	-1.368466
2.B	0.000000	0.000000	-1.725509
3.B	1.610322	0.775490	-1.368466
4.B	1.610322	-0.775490	-1.368466
5.B	0.397717	-1.742510	-1.368466
6.B	-1.114377	-1.397385	-1.368466
7.B	-1.787322	0.000000	-1.368466
8.B	-1.114377	1.397385	-1.368466
9.La	0.000000	0.000000	0.973581

II. (C_s, $^1A'$)

1.B	-0.384926	-1.495720	-1.633177
2.B	-1.706463	-1.300187	-0.800057
3.B	-1.706463	-1.300187	0.800057
4.B	-0.382605	-1.954316	0.000000
5.B	-0.384926	-1.495720	1.633177
6.B	1.081081	-1.457699	0.981421
7.B	2.036597	-0.589874	0.000000
8.B	1.081081	-1.457699	-0.981421
9.La	0.029057	0.780357	0.000000

LaB_8

I. (C_{7v}, $^2E_2$)

1.B	0.397461	1.741390	-1.303408
2.B	0.000000	0.000000	-1.682324
3.B	1.609287	0.774992	-1.303408
4.B	1.609287	-0.774992	-1.303408
5.B	0.397461	-1.741390	-1.303408
6.B	-1.113661	-1.396487	-1.303408
7.B	-1.786174	0.000000	-1.303408
8.B	-1.113661	1.396487	-1.303408
9.La	0.000000	0.000000	0.934065

II. (C_s, $^2A'$)

1.B	-0.361216	-1.455987	-1.594447
2.B	-1.701557	-1.378058	-0.787338
3.B	-1.708423	-1.373356	0.777828
4.B	-0.333290	-2.046045	0.003549
5.B	-0.374738	-1.447745	1.597466
6.B	1.094726	-1.445348	0.895059
7.B	2.071279	-0.522630	0.010293
8.B	1.103340	-1.447218	-0.881851
9.La	0.039624	0.797694	0.001153

PrB_8^-

I. (C_{7v}, $^5A_2$)

1.B	1.277862	1.116696	-1.393607
2.B	1.385194	1.783332	0.000000
3.B	1.277862	1.116696	1.393607

4.B	1.352036	-0.399967	1.740473
5.B	1.293955	-1.603981	0.781442
6.B	1.293955	-1.603981	-0.781442
7.B	1.691335	-0.005446	0.000000
8.B	1.352036	-0.399967	-1.740473
9.Pr	-0.938429	-0.000046	0.000000

II. (C_s, ^{3}A')

1.B	-1.414901	1.486454	0.798519
2.B	-0.083610	1.410128	1.629706
3.B	1.338500	1.066795	0.975970
4.B	2.058868	-0.006312	0.000000
5.B	1.338500	1.066795	-0.975970
6.B	-0.083610	1.410128	-1.629706
7.B	0.013887	1.874060	0.000000
8.B	-1.414901	1.486454	-0.798519
9.Pr	-0.152004	-0.829186	0.000000

PrB_8

I. (C_{7v}, $^4A_2$)

1.B	0.773632	1.609382	-1.276834
2.B	-0.000006	0.001285	-1.649409
3.B	1.731764	0.397209	-1.334685
4.B	1.390636	-1.107767	-1.311747
5.B	-0.000002	-1.783006	-1.239023
6.B	-1.390683	-1.107791	-1.311271
7.B	-1.731770	0.397211	-1.334904
8.B	-0.773628	1.609346	-1.277445
9.Pr	0.000004	-0.001240	0.929261

II. (C_s, ^{2}A')

1.B	-1.414904	1.486440	0.798505
2.B	-0.083596	1.410145	1.629700
3.B	1.338504	1.066790	0.975969
4.B	2.058860	-0.006318	0.000000
5.B	1.338504	1.066790	-0.975969
6.B	-0.083596	1.410145	-1.629700
7.B	0.013871	1.874083	0.000000
8.B	-1.414904	1.486440	-0.798505
9.Pr	-0.152004	-0.829187	0.000000

TbB_8^- (C_{7v}, $^7E_2$)

1.B	-0.396053	1.737708	-1.455159
2.B	-1.604771	0.771955	-1.450247
3.B	-1.606639	-0.773799	-1.455414
4.B	-0.000246	0.000041	-1.774774
5.B	-0.396819	-1.736521	-1.452436
6.B	1.110884	-1.393318	-1.458126
7.B	1.780677	-0.001030	-1.445306
8.B	1.109230	1.393145	-1.454661
9.Tb	0.000259	0.000126	0.849374

TbB_8 (C_{7v}, $^8E_2$)

1.B	-0.395526	1.734071	-1.375903
2.B	-1.601920	0.774669	-1.362188
3.B	-1.599099	-0.770439	-1.351886

4.B	0.000832	0.000316	-1.758799
5.B	-0.392898	-1.733932	-1.379274
6.B	1.110245	-1.390107	-1.369346
7.B	1.781430	-0.000794	-1.364543
8.B	1.108782	1.389595	-1.373106
9.Tb	-0.000821	-0.000234	0.807043

TmB_8^- (C_{7v}, $^3A_1$)

1.B	1.111525	-1.393809	-1.405927
2.B	1.782748	0.000000	-1.405927
3.B	1.111525	1.393809	-1.405927
4.B	0.000000	0.000000	-1.760164
5.B	-0.396699	1.738051	-1.405927
6.B	-1.606201	0.773505	-1.405927
7.B	-1.606201	-0.773505	-1.405927
8.B	-0.396699	-1.738051	-1.405927
9.Tm	0.000000	0.000000	0.837322

TmB_8 (C_{7v}, $^2A_1$)

1.B	1.110605	-1.392655	-1.306627
2.B	1.781272	0.000000	-1.306627
3.B	1.110605	1.392655	-1.306627
4.B	0.000000	0.000000	-1.705410
5.B	-0.396370	1.736612	-1.306627
6.B	-1.604871	0.772865	-1.306627
7.B	-1.604871	-0.772865	-1.306627
8.B	-0.396370	-1.736612	-1.306627
9.Tm	0.000000	0.000000	0.788455

YbB_8^- (C_{7v}, $^2A_1$)

1.B	-0.397793	1.742843	-1.448448
2.B	-1.610629	0.775638	-1.448448
3.B	-1.610629	-0.775638	-1.448448
4.B	0.000000	0.000000	-1.724387
5.B	-0.397793	-1.742843	-1.448448
6.B	1.114590	-1.397651	-1.448448
7.B	1.787663	0.000000	-1.448448
8.B	1.114590	1.397651	-1.448448
9.Yb	0.000000	0.000000	0.843652

YbB_8 (C_{7v}, $^1A_1$)

1.B	-0.397616	1.742070	-1.334325
2.B	-1.609915	0.775294	-1.334325
3.B	-1.609915	-0.775294	-1.334325
4.B	0.000000	0.000000	-1.657953
5.B	-0.397616	-1.742070	-1.334325
6.B	1.114095	-1.397032	-1.334325
7.B	1.786870	0.000000	-1.334325
8.B	1.114095	1.397032	-1.334325
9.Yb	0.000000	0.000000	0.788884

Supplementary Table 7. Theoretical VDE_1 and ADE values (eV) for the LnB_8^- clusters obtained from the PBE/TZP, PBE0/TZP and CCSD(T)/def2-TZVP methods, in comparison with the experimental data. All CCSD(T) values are single-point energies at fully optimized geometries obtained at the PBE0/TZP level.

	VDE_1				ADE			
	PBE	PBE0	CCSD(T)	Exp.	PBE	PBE0	CCSD(T)	Exp.
LaB_8^- (I)	1.45	1.42	1.47	~1.5	1.45	1.36	1.41	~1.35
LaB_8^- (II)	2.38	2.31	2.47	2.40	2.31	2.19 [a]	2.25	2.19
PrB_8^- (I)	1.43	1.55	1.54	~1.5	1.41	1.52	1.51	~1.4
PrB_8^- (II)	2.24	2.26	2.43	2.48	1.83	1.79	2.10	2.22
TbB_8^-	2.18	2.05	/	1.98	2.12	1.93	/	1.87
TmB_8^-	1.96	2.09	1.93	2.02	1.81	1.90	1.83	1.90
YbB_8^-	1.97	1.89	1.92	2.03	1.83	1.76	1.80	1.92

[a]This value was obtained from a PBE0/TZP single-point calculation based on the optimized neutral LaB_8 at the PBE/TZP level due to difficulties of the geometrical convergence at the PBE0 level.

Supplementary Table 8. Excitation energies (ΔE, eV) of different electron configurations of C_{7v} LnB_8^- (Ln = La, Pr, Tb, Tm, Yb) with Ln (+I) OS at the TD-PBE/TZP level.

Species	Electron configuration	ΔE
LaB_8^-	$6s^1 5d^1$	0.00
	$6s^2$	0.39
	$5d^2$	0.76
	$4f^1 6s^1$	0.85
	$4f^1 5d^1$	1.02
PrB_8^-	$4f^3 6s^1$	0.00
	$4f^2 6s^1 5d^1$	0.19
	$4f^2 6s^2$	0.25
	$4f^3 5d^1$	0.68
	$4f^4$	1.13
TbB_8^-	$4f^8 6s^2$	0.00
	$4f^8 6s^1 5d^1$	0.18
	$4f^9 6s^1$	0.44
	$4f^7 6s^2 5d^1$	0.59
TmB_8^-	$4f^{13} 6s^1$	0.00
	$4f^{12} 6s^2$	0.21
	$4f^{14}$	0.77
	$4f^{13} 5d^1$	1.60
	$4f^{12} 6s^1 5d^1$	1.91
YbB_8^-	$4f^{14} 6s^1$	0.00
	$4f^{13} 6s^2$	0.41
	$4f^{13} 6s^1 5d^1$	1.40

Supplementary Table 9. EDA results for the C_{7v} and C_s isomers of LnB_8^- (Ln = La, Pr, Tb, Tm, Yb) at the PBE0/TZP level with the atomic fragments. Relative energies in each term are in kcal/mol with the C_{7v} isomer as the reference.

Energy term		LaB_8^-	PrB_8^-	TbB_8^-	TmB_8^-	YbB_8^-
$\Delta E_{tot}{}^{a}$	C_{7v}	0.00	0.00	0.00	0.00	0.00
	C_s	-3.87	-4.32	+9.17	+25.22	+19.65
ΔE_{pauli}	C_{7v}	0.00	0.00	0.00	0.00	0.00
	C_s	+80.70	+66.20	+29.55	+58.45	+84.27
ΔE_{elstat}	C_{7v}	0.00	0.00	0.00	0.00	0.00
	C_s	-112.43	-113.55	-102.20	-94.00	-142.55
$\Delta E_{steric}{}^{b}$	C_{7v}	0.00	0.00	0.00	0.00	0.00
	C_s	-31.73	-47.35	-72.65	-35.55	-58.28
ΔE_{orb}	C_{7v}	0.00	0.00	0.00	0.00	0.00
	C_s	+27.86	+43.04	+81.88	+60.83	+77.49

[a] $\Delta E_{tot} = \Delta E_{steric} + \Delta E_{orb}$.

[b] $\Delta E_{steric} = \Delta E_{pauli} + \Delta E_{elstat}$.

Supplementary Table 10. Orbital compositions of the $2e_1$ molecular orbital with the metal-B_8 π type of bonding character at the PBE0/TZP level.

	$2e_1$	
	Ln	B
LaB_8^-	12.7% 5d	81.1% 2p + 3.9% 2s
PrB_8^-	10.2% 5d	89.7% 2p
TbB_8^-	8.1% 5d	90.0% 2p + 1.8% 2s
TmB_8^-	4.8% 5d + 1.8% 6p	82.2% 2p + 4.1% 2s
YbB_8^-	4.1% 5d + 4.0% 4f	92.0% 2p + 2.2% 2s

Supplementary Table 11. The EDA-NOCV analysis of the C_{7v} LnB_8^- (Ln = La, Pr, Tb, Tm, Yb) at the PBE/TZ2P level. Only major contribution (magnitude >10 kcal/mol) to the orbital interaction was listed. Charge flows from red to blue. Energy values are given in kcal/mol.

	LaB_8^-		PrB_8^-	
Fragment	La^+ ($5d^16s^1$) + B_8^{2-} ($\ldots 2e_1^4 1e_2^0$)		Pr^+ ($4f^36s^1$) + B_8^{2-} ($\ldots 2e_1^4 1e_2^0$)	
Spin	α	β	α	β
$\Delta E_{orb(1)}$	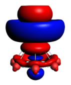 -40.5		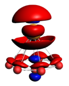 -26.8	
$\Delta E_{orb(2)}$	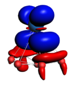 -18.1	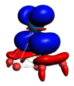 -15.9	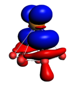 -17.6	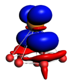 -15.2
$\Delta E_{orb(2)'}$	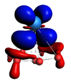 -18.1	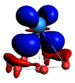 -15.9	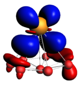 -17.6	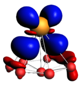 -15.2
$\Delta E_{orb(3)}$	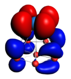 -10.8			

	TbB_8^-	
Fragment	Tb^+ ($4f^86s^2$) + B_8^{2-} ($\ldots 2e_1^4 1e_2^0$)	
Spin	α	β
$\Delta E_{orb(1)}$	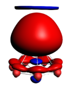 -23.5	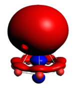 -59.7
$\Delta E_{orb(2)}$	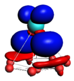 -16.8	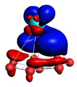 -14.7
$\Delta E_{orb(2)'}$	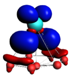 -16.8	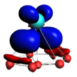 -14.7

	TmB_8^-		YbB_8^-	
Fragment	Tm^+ ($4f^{13}6s^1$) + B_8^{2-} ($\ldots 2e_1^4 1e_2^0$)		Yb^+ ($4f^{14}6s^1$) + B_8^{2-} ($\ldots 2e_1^4 1e_2^0$)	
Spin	α	β	α	β
$\Delta E_{orb(1)}$	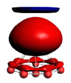 -23.3		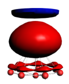 -22.7	
$\Delta E_{orb(2)}$	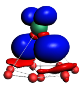 -11.3	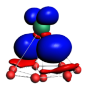 -10.5	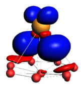 -10.1	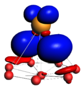 -9.6
$\Delta E_{orb(2)'}$	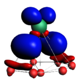 -11.3	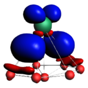 -10.5	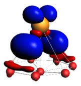 -10.1	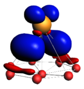 -9.6

Supplementary Table 12. EDA-NOCV results for C_{7v} LnB_8^- (Ln = La, Pr, Tb, Tm, Yb) at the PBE/TZ2P level using the PBE0/TZP optimized geometries, taking the interacting fragments Ln^+ and B_8^{2-} with the same electronic configuration as that in the cluster. Energies are in kcal/mol.

Energy term	LaB_8^-	PrB_8^-	TbB_8^-	TmB_8^-	YbB_8^-	KB_8^-
ΔE_{int}	-284.1	-291.3	-234.3	-255.4	-252.3	-198.0
ΔE_{pauli}	204.2	201.5	176.6	139.5	126.7	29.9
ΔE_{elstat}	-335.5	-324.8	-286.4	-290.9	-280.9	-199.7
	(68.7%)[a]	(65.9%)	(69.7%)	(73.7%)	(74.1%)	(87.6%)
ΔE_{orb}	-152.8	-167.9	-124.5	-104.0	-98.2	-28.2
	(31.3%)[a]	(34.1%)	(30.3%)	(26.3%)	(25.9%)	(12.4%)

[a] The values in parentheses show the contribution to the total attractive interactions ΔE_{elstat} plus ΔE_{orb}, where ΔE_{elstat} is the electrostatic interaction energy.

Supplementary Table 13. LnB_n^- species with different boron ligands, different oxidation state (OS) of Ln with selected examples.

B_n^-	B_7^{3-}	B_8^{2-}	B_9^-	
OS	Ln (II)	Ln (I)	Ln (0)	
Example	PrB_7^-	LaB_8^-, PrB_8^-, TbB_8^-, TmB_8^-, YbB_8^-	LnB_9^- (?)	/

Supplementary Table 14. Direct products of representations of the C_{7v} point-group symmetry.

	A_1	A_2	E_1	E_2	E_3
A_1	A_1	A_2	E_1	E_2	E_3
A_2		A_1	E_1	E_2	E_3
E_1			$A_1 \oplus \{A_2\} \oplus E_2$	$E_1 \oplus E_3$	$E_2 \oplus E_3$
E_2				$A_1 \oplus \{A_2\} \oplus E_3$	$E_1 \oplus E_2$
E_3					$A_1 \oplus \{A_2\} \oplus E_1$
